# The pathogenesis, detection, and prevention of *Vibrio parahaemolyticus*

**DOI:** 10.3389/fmicb.2015.00144

**Published:** 2015-03-05

**Authors:** Rongzhi Wang, Yanfang Zhong, Xiaosong Gu, Jun Yuan, Abdullah F. Saeed, Shihua Wang

**Affiliations:** Key Laboratory of Biopesticide and Chemical Biology of Education Ministry and Key Laboratory of Pathogenic Fungi and Mycotoxins of Fujian Province, School of Life Sciences, Fujian Agriculture and Forestry UniversityFuzhou, China

**Keywords:** *Vibrio parahaemolyticus*, pathogenesis, virulence factor, detection, prevention

## Abstract

*Vibrio parahaemolyticus*, a Gram-negative motile bacterium that inhabits marine and estuarine environments throughout the world, is a major food-borne pathogen that causes life-threatening diseases in humans after the consumption of raw or undercooked seafood. The global occurrence of *V. parahaemolyticus* accentuates the importance of investigating its virulence factors and their effects on the human host. This review describes the virulence factors of *V. parahaemolyticus* reported to date, including hemolysin, urease, two type III secretion systems and two type VI secretion systems, which both cause both cytotoxicity in cultured cells and enterotoxicity in animal models. We describe various types of detection methods, based on virulence factors, that are used for quantitative detection of *V. parahaemolyticus* in seafood. We also discuss some useful preventive measures and therapeutic strategies for the diseases mediated by *V. parahaemolyticus*, which can reduce, to some extent, the damage to humans and aquatic animals attributable to *V. parahaemolyticus*. This review extends our understanding of the pathogenic mechanisms of *V. parahaemolyticus* mediated by virulence factors and the diseases it causes in its human host. It should provide new insights for the diagnosis, treatment, and prevention of *V. parahaemolyticus* infection.

*Vibrio parahaemolyticus,* a kind of Gram-negative motile bacteria inhabiting marine and estuarine environments throughout the world (Wang et al., 2011a), is a major food-borne pathogen that causes diarrhea primarily after the consumption of raw or undercooked seafood (Bresee et al., 2002; Kawatsu et al., 2006). To ensure its survival in varying environments, *V. parahaemolyticus* has two different types of flagellar systems, allowing it to adapt to constantly changing environments. The polar flagellum is responsible for swimming (Broberg et al., 2011), whereas the lateral flagella are closely associated with the swarmer cell type transformation and biofilm formation (**Figure 1**). During infection, *V. parahaemolyticus* uses the adhesion factors to bind to the fibronectin and phosphatidic acid on the host cell, thus releasing different effectors and toxins into the cytoplasm, causing cytotoxicity and serious diseases (Gode-Potratz et al., 2011). *V. parahaemolyticus* is a multiserotype bacterium, containing at least 12 different O antigens and more than seventy different K antigens in its capsule. Consequently, serotyping has been widely used to detect *V. parahaemolyticus* and to study its pathogenesis (Xu et al., 2014). Among the serotypes, three serotypes (O3:K6, O4:K68, and O1:K untypeable) are extremely virulent and pathogenic to humans, and are regarded as the major agents of food-borne diseases (Jones et al., 2012). To date, the genomes of six strains from these different serotypes have been sequenced: strains RimD221063 and AQ3810 from O3:K6, the strains AN-5034, K5030, and Peru-466 from O4:K68, and the strain Vp10329 from O4:K12 (Makino et al., 2003; Broberg et al., 2011; Gonzalez-Escalona et al., 2011). The first fully sequenced and annotated genome of strain RimD221063 has been used as the reference sequence in cell biological and pathogenetic analysis of numerous clinical and environmental *V. parahaemolyticus* strains (Makino et al., 2003).

**FIGURE 1:**
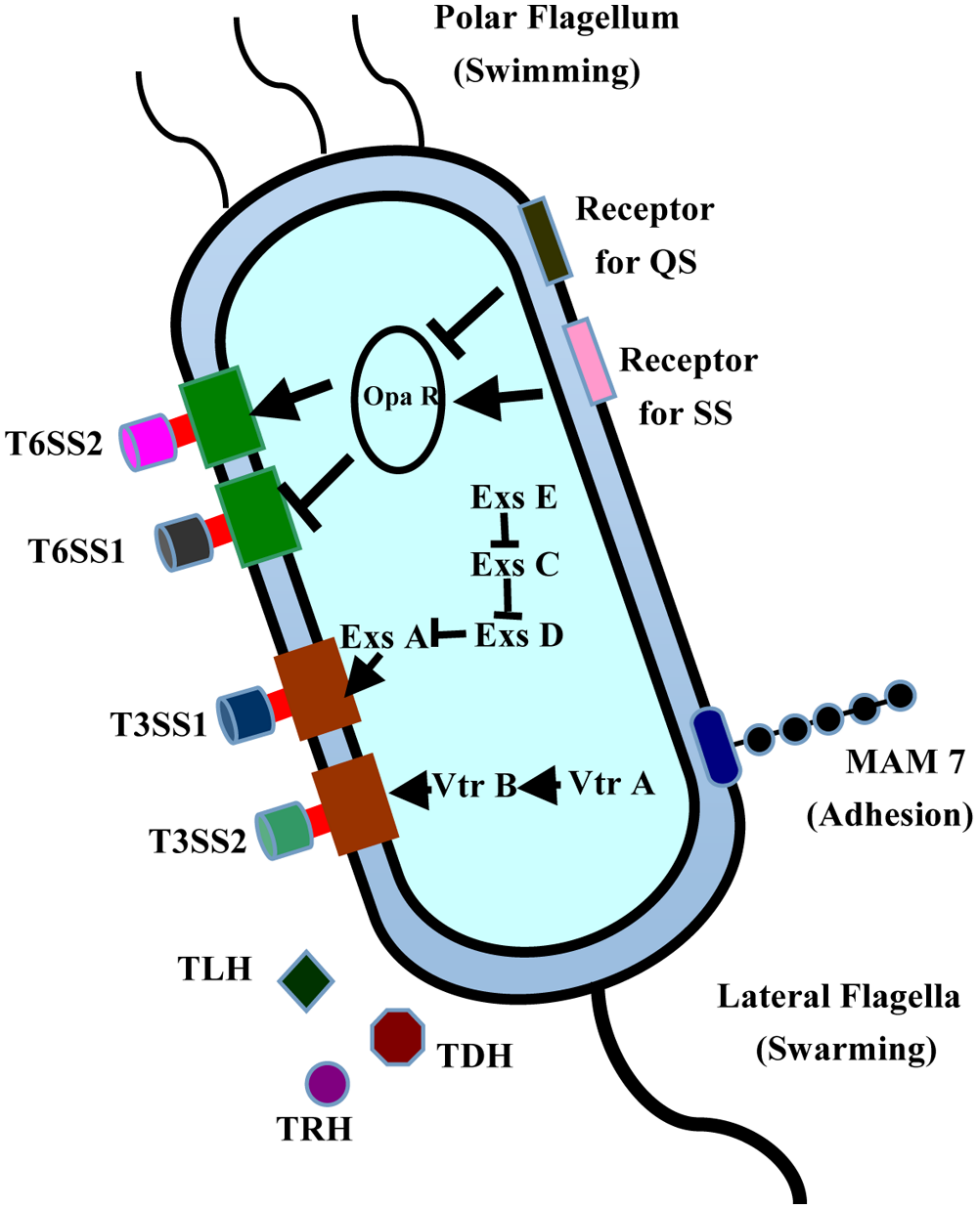
**Structures and virulence factors of *V. parahaemolyticus*.** *V. parahaemolyticus* contains two T3SS systems, two T6SS systems, and expresses many toxins, including TLH, TRH, and TDH. MAM7 is responsible for its initial attachment to host cells. This bacterium has two different flagellar systems, allowing it to adapt to changing environments. The polar flagellum is responsible for swimming, whereas the lateral flagella are closely related to the swarmer cell transformation and biofilm formation.

## DISEASE CAUSED BY *V. parahaemolyticus*

In 1950, an first outbreak of disease caused by *V. parahaemolyticus* in the city of Osaka city of Japan was first reported, with acute gastroenteritis in 272 individuals, 20 of whom died (Fujino et al., 1953; Daniels et al., 2000). Since then, 802 outbreaks of food-borne diseases have been reported in 13 of the coastal provinces of eastern China, causing 17,462 individuals to become ill (Wang et al., 2011a). *V. parahaemolyticus* (40.1%) accounted for the greatest number of these outbreaks and cases (Liu et al., 2006; Chao et al., 2010). Similar diseases have also been frequently reported in many countries in Asia, Europe, Africa, and in the Americans (DePaola et al., 2000; Alam et al., 2002; Lozano-León et al., 2003; Martinez-Urtaza et al., 2005; Su and Liu, 2007; Chao et al., 2009). *V. parahaemolyticus* infection is also disseminated through open wounds, and often causes septicemia in severe cases (Wang et al., 2012). Recently, *V. parahaemolyticus* has been reported to be the major agent of acute hepatopancreatic necrosis syndrome aﬄicting penaeid shrimp, and has seriously damaged the shrimp aquaculture industry (Tran et al., 2013).

The food poisoning caused by *V. parahaemolyticus* usually occurs in summer (from June to October), and is predominantly associated with different kinds of seafood, including crab, shrimp, shellfish, lobster, fish, and oysters (Lee et al., 2008; Nakaguchi, 2013; Jones et al., 2014; Cruz et al., 2015; Letchumanan et al., 2015). Among the whole range of seafood, shellfish is regarded as a high-risk food because it is infested with large populations of bacteria, including *V. parahaemolyticus,* to levels higher than those in the surrounding water (Peng et al., 2010; Anonymous, 2012). Once consumers eat undercooked, contaminated seafood, illness is inevitable (Rahimi et al., 2010). The typical clinical symptoms of *V. parahaemolyticus* poisoning are acute dysentery and abdominal pain, accompanied by diarrhea, nausea, vomiting, fever, chills, and water-like stools (Yeung and Boor, 2004; Shimohata and Takahashi, 2010). The feces of patients are mixed with mucus or blood, and their blood pressure decreases dreamily, leading to shock (Broberg et al., 2011). Some severely affected patients become unconsciousness, show recurrent convulsions, become pale or cyanotic, and even death (Nair et al., 2007). The distinct pathological changes in patients include the mild erosion of the jejunum and ileum, gastric inflammation, and internal organ damage (liver, spleen, lung congestion, etc.). To cure *V. parahaemolyticus* infection effectively, common treatment methods include antibiotics and oral rehydration. To avoid intense illness, it is recommended that some subpopulations, including patients suffering severe physical or immunodeficiency diseases, do not consume the seafood (Blake et al., 1979; Hlady and Klontz, 1996).

## PATHOGENESIS OF *V. parahaemolyticus*

### HEMOLYSIN, UREASE, AND PATHOGENESIS

Thermostable direct hemolysin (TDH) and TDH-related hemolysin (TRH) are two major virulence factors of *V. parahaemolyticus*, and are closely related to its pathogenicity (**Table 1**). They have similar hemolytic activity *in vitro* and cause the lysis of human erythrocytes in excessively saline medium (Sakazaki et al., 1968; Miyamoto et al., 1969; Hondo et al., 1987; Honda et al., 1988). An epidemiological investigation indicated that TDH is one of the major pathogenic factors in *V. parahaemolyticus*, and is prevalent in almost all (95%) of clinical isolates. When secreted, it can lyse red blood cells and produces a special hemolysis ring on Wagatsuma blood agar plates (Nishibuchi et al., 1992; Honda and Iida, 1993; Liu, 2003). This is also known as “Kanagawa phenomenon” and is reported to be commonly associated with gastroenteritis (Miyamoto et al., 1969; Joseph et al., 1982). Previous reports have shown that two enzymatic activities of TDH are associated with bacterial pathogenesis. One is a hemolytic activity that is independent of lipid rafts. TDH binds to the membranes of red blood cells or host cells, and forms a pore on the membrane surface, ultimately leading to the permeation of the colloids of red blood cells (Matsuda et al., 2010). The other enzymatic activity is its cytotoxicity, TDH causes cells toxicity and forms a channel in the cell membrane, which induces an increase in the extracellular Ca^2+^ concentration and Cl^-^ secretion (Matsuda et al., 2010). When the osmotic pressure of the cell exceeds the upper limit for cell self-regulation, pathological and morphological changes were occur in the cell, resulting in cell expansion and even death. Like TDH, TRH causes similar levels of hemolysis *in vitro* (Takahashi et al., 2000; Ceccarelli et al., 2013).

**Table 1:** List of known virulence factors of *V. parahaemolyticus.*

Effectors	Gene	Biological activity	Effects on host cells
**Toxins**
TDH	*tdh*	Forms pores on cells	Cytotoxicity and enterotoxicity
TRH	*trh*	Forms pores on cells	Cytotoxicity and enterotoxicity
TLH	*tlh*	Hemolysin activity or?	Cytotoxicity and ?
**T3SS1 effectors**
Vop Q	*vp1680*	Forms pores and binds V-ATPase	Autophagy, cell lysis, MAPK activation, IL-8 secretion
Vop S	*vp1686*	Inhibition of Rho by AMPylation	Cells rounding, phagocytes invasion
VPA0450	*vpa0450*	Phosphatidylinositol phosphatase	Membrane blebbing, destabilization
Vop R	*vp1683*	Binds PIP2 in membrane	Promoting refolding of T3SS effectors
**T3SS2 effectors**
Vop A/P	*vpa1346*	Inhibition of MAPK by acetylation of MKK	Blocking of phosphorylation and ATP binging
Vop T	*vpa1327*	Ras ADP-ribosylation	Cytotoxicity and yeast growth inhibition
Vop L	*vpa1370*	Actin nucleation	Stress fibers formation and cell shape changing
Vop C	*vpa1321*	Activation of Rac and CDC42 by deamidation	Invasion of non-phagocytic cells
Vop V	*vpa1357*	Actin binding and bundling	Enterotoxicity and blunting of villi
Vop Z	*vpa1336*	Inhibition of TAK1 and downstream MAPK and NF-_k_B	Enterotoxicity and colonization
VPA1380	*vpa1380*	Cysteine catalysis dependent on IP6	Toxicity in yeast

Thermolabile hemolysin (TLH) is another hemolysin of *V. parahaemolyticus*, encoded by the *tlh* gene, and also causes the lysis of red blood cells (Shinoda et al., 1991; McCarthy et al., 1999; Wang et al., 2013b). TLH is expressed by all clinical and environmental strains of *V. parahaemolyticus* (Bej et al., 1999), and the gene is significantly upregulated under simulated intestinal infection conditions (Gotoh et al., 2010; **Table 1**). Besides, TLH shows typical lecithin-dependent phospholipase activity, and it also lyses human erythrocytes (Broberg et al., 2011). Therefore, it may play a key role in the process of human infection. Recent, studies have demonstrated that all three types of cells (Hela, Changliver, and RAW264.7 cells) display signs of severe cytotoxicity when treated with the purified TLH protein, and its effects are dose and time-dependent (Wang et al., 2012). Therefore, TLH may have similar biological functions similar to these of the TDH and TRH toxins, playing a key role in the *V. parahaemolyticus* infection. Early studies showed that urease induces the accumulation of intestinal fluid in the rabbit ileal loops test and causes gastrointestinal inflammatory lesions, confirming that urease is an important virulence factor in *trh*^+^ *V. parahaemolyticus* strains (Cal and Ni, 1996; Osawa et al., 1996). Urease is encoded by the *Uh* gene, and is generally involved in the formation of ammonia during the process of infection (Levin, 2006).

### T3SS1 INDUCES AUTOPHAGY AND CYTOTOXICITY

**FIGURE 2:**
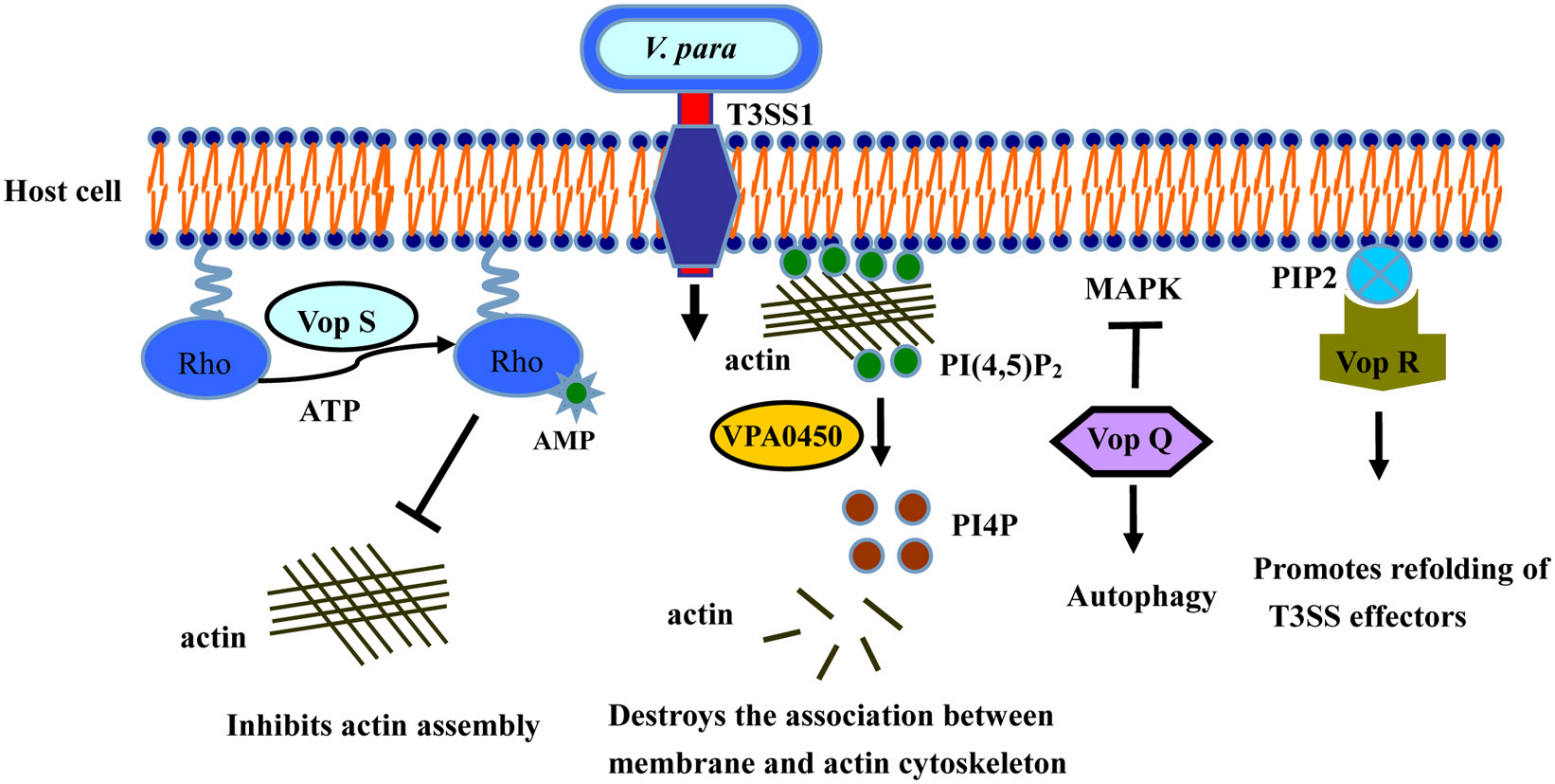
**Activities of T3SS1 effectors in cultured intestinal epithelial cells.** Vop Q inhibits the MAPK pathway by acetylating MKK, and Vop S inhibits Rho by AMPylation, leading to cells rounding and phagocytes invasion. VPA0450 hydrolyzes phosphatidylinositide (4, 5)-bisphosphate (PI(4,5)P2) to D5 phosphate (PI4P) and disrupts the association between the membrane and the actin cytoskeleton, leading to membrane blebbing. Vop R binds to PIP2 in the membrane, thus promoting the refolding of the T3SS effectors.

The type III secretion systems (T3SSs) are transmembrane apparatuses formed by the multicomponent protein complexes (Cornelis, 2006), that allow effectors or virulence proteins to be injected directly into the cytoplasm of the host cell (Dean, 2011; Chatterjee et al., 2013). There are two different T3SS systems in *V. parahaemolyticus*, designated T3SS1 and T3SS2 (Makino et al., 2003). T3SS1 is located on chromosome I, is encoded by the first pathogenicity island, and is present in almost every clinical and environmental *V. parahaemolyticus* strains (Paranjpye et al., 2012). T3SS1 gene expression is regulated by three exoenzyme S synthesis proteins (ExsC, ExsD, and ExsE) and heat-stable nucleoid structuring protein (H-NS; Kodama et al., 2010; Zhou et al., 2010). Several studies have shown that T3SS1 is cytotoxic, causing autophagy, cell rounding, and finally death (Burdette et al., 2008; Hiyoshi et al., 2010; Okada et al., 2010; Ritchie et al., 2012; Zhang and Orth, 2013). To date, four effectors have been determined in T3SS1 (**Table 1**): Vop Q, Vop S, VPA0450, and Vop R (VP1638), correspondingly (Broberg et al., 2010; Luong et al., 2010; Salomon et al., 2013a; Sreelatha et al., 2013; **Figure 2**).

The effector Vop Q (Park et al., 2004a) is necessary for the formation of autophagic vesicles in the process of *V. parahaemolyticus* infection (Matsuda et al., 2012). Many researchers have confirmed that the *V. parahaemolyticus* strain in which T3SS1 is deleted can be easily engulfed and degraded by macrophages, causing the apoptosis of the infected cells (Burdette et al., 2009; Sreelatha et al., 2013). These results indicate that T3SS1 effectively inhibits the ability of the host cells to phagocytose *V. parahaemolyticus* (Jegga et al., 2011). Vop Q is also reported to be an activator of the JNK, p38, and MAPK pathways in Caco-2 cells, and interacts with the C subunit of the vacuolar H^+^-ATPase (Matlawska-Wasowska et al., 2010; Porta et al., 2011), leading to the secretion of the chemokine interleukin 8 (IL-8; Shimohata et al., 2011).

Vop S, another effector secreted by T3SS1, causes the death of macrophages by inhibiting NF-κB activity. Vop S contains a Fic domain at its C terminus, and also prevents actin aggregation and rapid re-aggregation by AMPylating the Rho family GTPases (Casselli et al., 2008; Yarbrough et al., 2009; Luong et al., 2010), resulting in the rounding of the infected cells (Kim and Jo, 2013). This change allows the pathogen to suppress the phagocytosis of infected cells by macrophages (Higa et al., 2013). Vop S also has exerts certain cytotoxicity effects on Hela cells.

VPA0450 is a typical phosphatidylinositide phosphatase, hydrolyzing phosphatidylinositide (4, 5)-bisphosphate (PI(4,5)P2) to D5 phosphate (PI4P; Krauss and Haucke, 2007). The resulting product, PI4P, disrupts the association between the membrane and the actin cytoskeleton, leading to membrane blebbing (Broberg et al., 2010). VPA0450 induces cell rounding and lysis by destroying the dynamics of the plasma membrane cytoskeleton, and it may play a complementary role with other effectors in the infection process (Broberg et al., 2010).

Vop R is encoded by the *vp1683* gene and is secreted by T3SS1. It also contains a similar phosphoinositide-binding domain (BPD) that is conserved in diverse type III effectors of both plant and animal pathogens (Salomon et al., 2013a). Vop R localizes to the host membrane by its N-terminal domain and specifically binds the phosphoinositide on the host cell. It may also play a key role in promoting the refolding of Type III effectors after their delivery into the host cells (Geissler, 2012; Hicks and Galan, 2013; Salomon et al., 2013a).

### T3SS2 MEDIATES ENTEROTOXICITY AND CYTOTOXICITY

**FIGURE 3:**
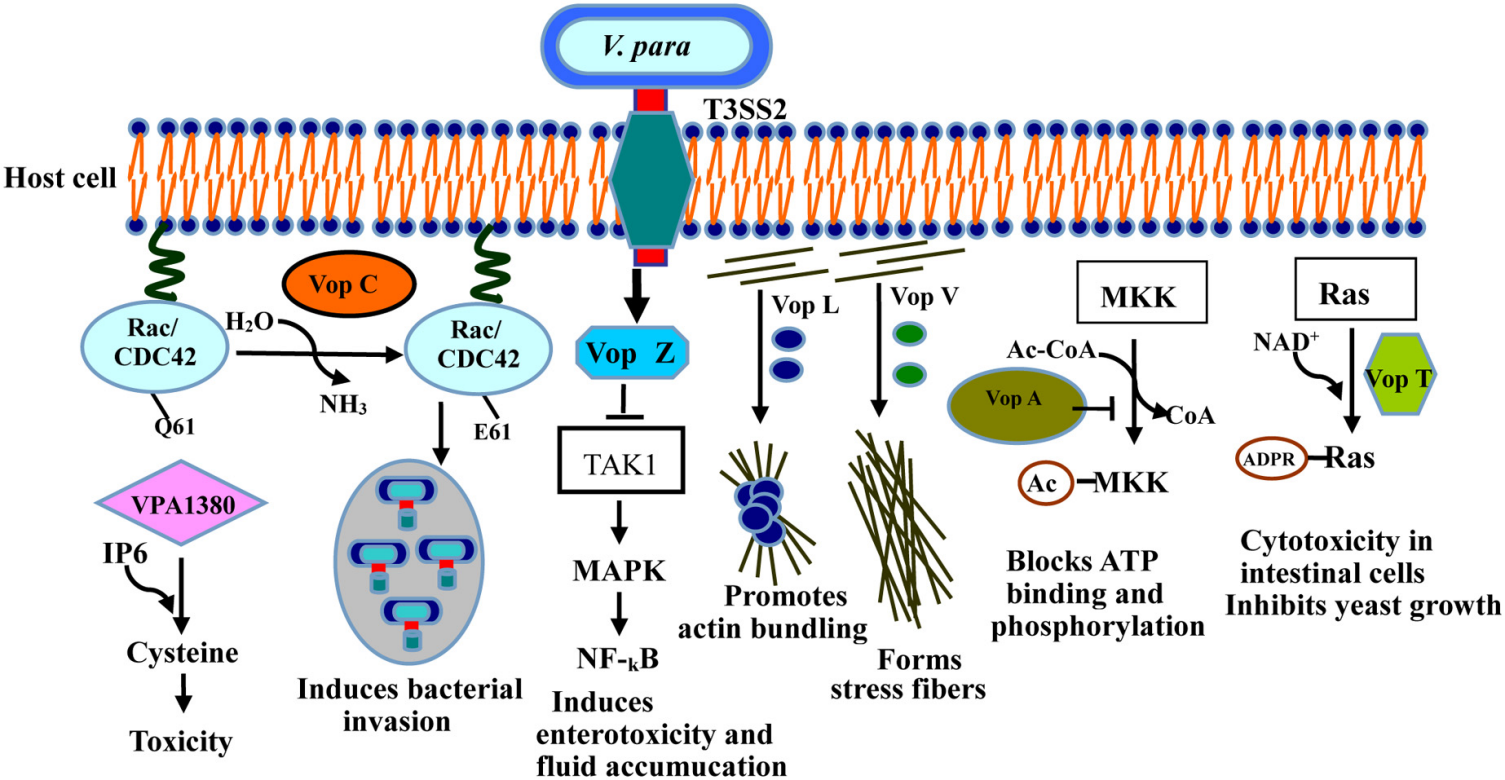
**Activities of the T3SS2 effectors in intestinal epithelial cells during infection.** Vop C deamidates small GTPases such as Rac and CDC42, inducing bacterial invasion. Vop L dimerizes through its VCD domain, thus promoting actin bundling. Vop A inhibits MAPK by acetylating of MKK. Vop V binds directly to F-actin through its long repeat (LR) and C-terminal domain, forming the stress fibers. Vop T modifies Ras with ADP-ribose, and triggers yeast growth inhibition and cytotoxicity in intestinal cells. Vop Z inhibits the activation of the MAPK and NF-κB pathways by repressing of TAK1 kinase. VPA1380 catalyzes it targeted substrate.

T3SS2, a newly identified type of secretion system, is encoded by a pathogenicity island (*VP-PAL*) on chromosome II, and is found primarily in clinical isolates (Meador et al., 2007). T3SS2 has been associated with enterotoxicity in the rabbit ileal loop model, infant rabbits and piglets (Makino et al., 2003; Paranjpye et al., 2012), and has also been shown to cause cytotoxicity in intestinal cell lines such as Caco-2 cells and HCT cells (Park et al., 2004b; Hiyoshi et al., 2010; Ritchie et al., 2010; **Figure 3**). Seven known effectors have so far been identified and characterized in T3SS2 (**Table 1**): Vop C, Vop T, Vop Z, Vop A/P, Vop V, Vop L, and VPA1380 (Trosky et al., 2004; Kodama et al., 2007; Liverman et al., 2007; Yu et al., 2011; Zhang et al., 2012; Calder et al., 2014).

Vop A/P (VPA1346) is an acetyltransferase with 55% homology to Yop J of *Yersinia* spp. (Makino et al., 2003), which blocks the MAPKs signaling pathway by inhibiting the start and biological activity of mitogen-activated protein kinase (Trosky et al., 2004), thereby suppressing cell division *via* a new mechanism. Vop L (VPA1370) contains three Wiskott Aldrich homology 2 (WH2) domains and a C-terminal domain (VCD; Namgoong et al., 2011; Yu et al., 2011), which generally induces the formation of polarized actin fibers and accelerates the gathering of actin filaments by binding to actin monomers (Liverman et al., 2007). Notably, Vop L may provide a favorable microenvironment in which bacteria can replicate, thereby enhancing the uptake and invasion of *V. parahaemolyticus.*

Vop C disturbs the actin network and causes bacterial invasion by deamidating glutamine 61 in both Rac and CDC42, which occurs in their switch regions, resulting in the constitutive activation of the Rho family GTPases (Friebel et al., 2001; Zhang et al., 2012). The actin cytoskeletons of infected cells are further rearranged with the modification of these GTPases, thereby prompting the infected cells to engulf the bacteria (Zhang et al., 2012). Vop T modifies the small G protein with ADP-ribose using NAD^+^ as the substrate *in vivo* and *in vitro* (Fraylick et al., 2002; Barbieri and Sun, 2004). Vop T inhibits yeast cell growth and is cytotoxic for Caco-2 and HCT-8 cells (Kodama et al., 2007).

Vop V has long repeat (LR) regions in its N- and C-terminal domains, composed of three types of repeated sequence units. It predominantly induces the enterotoxicity observed in the rabbit ileal loop model (Hiyoshi et al., 2011), and binds directly to F-actin, a polymeric form of actin, leading to the accumulation of F-actin filaments beneath the bacterial microcolonies in Caco-2 cells (Haglund and Welch, 2011). All the above results indicate that F-actin binding is required for the enterotoxicity caused by Vop V (Hiyoshi et al., 2011). However, the molecular mechanism of this enterotoxicity is still unclear, so further investigations are needed for underlying its crucial role during infection.

Vop Z, a novel effector secreted by the T3SS2 system, is responsible for fluid accumulation, cell detachment, and epithelial damage (Zhou et al., 2013). Intestinal colonization by *V. parahaemolyticus* and fluid accumulation are reduced when Vop Z is deleted (Zhou et al., 2013). Vop Z inhibits the activation of the MAPK and NF-κB signaling pathways by inhibiting the activation of the TAK1 kinase, resulting in a marked lesion, disrupting on the integrity of the tissue (Kajino-Sakamoto et al., 2008; Zhou et al., 2013). Therefore, it plays a critical role in the virulence of *V. parahaemolyticus.*

VPA1380 was recently identified as a critical effector of *V. parahaemolyticus* translocated by T3SS2 (Hiyoshi et al., 2011). It was detrimental to and exerted a toxic effect on yeast growth when it was expressed as an enhanced green fluorescent protein (eGFP) fusion protein, when yeast was used as a heterologous eukaryotic system (Calder et al., 2014). Bioinformatic analyses revealed that VPA1380 contains several inositol hexakisphosphate (IP6)-inducible cysteine protease domains, which are known to occur in large known toxins produced by other bacteria (Prochazkova and Satchell, 2008; Pruitt et al., 2009). VPA1380 is reported to be a typical cysteine protease, catalyzing its targeted substrate (Calder et al., 2014), so VPA1380 is possibly involved in the invasion of host cells by *V. parahaemolyticus*.

### T6SS AND PATHOGENESIS

Recently, a type VI secretion system (T6SS) has been identified, and detected in many Gram-negative bacteria. It is a macromolecular machine consisting of a multicomponent protein complex (Ho et al., 2014). T6SS is responsible for delivering a series of toxic effector proteins into the cytoplasm of eukaryotic cells, allowing the effectors to disrupt the innate immune system and to kill the host cells (Coulthurst, 2013). The T6SS organelle is functionally analogous to T3SS, and may have a critical function in the process of bacterial infection (Salomon et al., 2013a). Interestingly, *V. parahaemolyticus* contains two different T6SS systems, designated T6SS1 and T6SS2 (Boyd et al., 2008; Yu et al., 2012). T6SS1 is encoded on chromosome I, is predominantly expressed in clinical isolates, and it is most active under warm conditions (O’Boyle and Boyd, 2014). T6SS2 has been found in both clinical and environmental isolates, is encoded on chromosome I, and is active under low-salt conditions (Salomon et al., 2013b). A homology analysis indicated that the T6SSs are present in most different *Vibrio* species, including *V. parahaemolyticus*, *V. cholerae, V. harveyi,* and *V. alginolyticus* (Pukatzki et al., 2006). Recently published medical research articles have reported that both T6SSs are necessary for the adhesion of *V. parahaemolyticus* to cells and are involved in intracellular trafficking and vesicular transport (Boyd et al., 2008; Yu et al., 2012; Salomon et al., 2013b). Only a few effectors of T6SS from *V. parahaemolyticus* have so far been reported. In recent research, two T6SS effectors that mediate its antibacterial activity were identified using proteomic, bioinformatic, and genetic analyses (Salomon et al., 2014). VP1388 is encoded within the T6SS1 gene cluster, whereas VPA1263 is encoded on chromosome II (Salomon et al., 2014). The two effectors contain the conserved MIX motif that is found in proteins with predicted cytotoxic domains, including VgrG and PAAR-repeat-containing protein (Pukatzki et al., 2007; Shneider et al., 2013).

## DETECTION METHODS BASED ON VIRULENCE FACTORS

### KANAGAWA TEST

Thermostable direct hemolysin is a virulence factor that contributes to the formation of a distinct hemolytic ring on blood cells agar plates in high concentrations of salt with D-mannitol as the carbon source, known as the “Kanagawa phenomenon” (KP; Honda and Iida, 1993; Nishibuchi and Kaper, 1995). In the past, the KP has been regarded as an important indicator in the identification of the pathogenic and non-pathogenic *V. parahaemolyticus* strains (Zhang and Austin, 2005; Ono et al., 2006). However, the detection of *V. parahaemolyticus* based on KP is time-consuming, labor intensive, and unreliable, and involves the evaluation of large numbers of samples (Park et al., 2004c; Wang et al., 2011b). Therefore, the development of specific, sensitive, and rapid methods to detect this bacterium is crucial for public health.

### PCR DETECTION

Polymerase chain reaction (PCR) assays are being increasingly used to identify and distinguish specific pathogenic bacteria. Multiplex PCR protocols targeting the *toxR*, *tlh*, *tdh*, *trh*, and *fla* genes have been developed to detect the total and pathogenic *V. parahaemolyticus* from clinical and environmental samples (Rosec et al., 2009; Izumiya et al., 2011; Wang et al., 2011a; Hossain et al., 2013). Recently, a serogroup-*O*-specific PCR assay was used to detect and identify *V. parahaemolyticus* pathogens in clinical and environmental samples (Chen et al., 2012). Before 2012, multiplex real-time PCR with different fluorescent probes was used to detect total and pathogenic *V. parahaemolyticus* in different kinds of seafood (Ward and Bej, 2006; Nordstrom et al., 2007; Tyagi et al., 2009; Robert-Pillot et al., 2010). Garrido used multiplex real-time PCR to detect pathogenic *V. parahaemolyticus* in water and food samples. The limits of detection for this method were 0.24 CFU/g for *tdh*, and 0.44 CFU/g for *trh1*, and 0.52 CFU/g for *trh2* (Garrido et al., 2012). A quantitative PCR method combined with propidium monoazide has also been used to quantify the viable *V. parahaemolyticus* cells in raw seafood (Zhu et al., 2012). In general, detection methods based on PCR are quick, high accuracy and sensitivity, but the main disadvantages of that is badly controllability, and the PCR system often need to be optimized to gain the best detection results (Letchumanan et al., 2014).

Loop-mediated isothermal amplification (LAMP) is a specific and highly sensitive technique for DNA amplification under isothermal conditions with the specific primers, and has been widely used to detect pathogenic bacteria in food (Zhao et al., 2011; Qi et al., 2012). LAMP targeting the *tlh*, *tdh*, or *toxR* genes of *V. parahaemolyticus* is used for the sensitive and rapid detection of *V. parahaemolyticus* (Yamazaki et al., 2008; Nemoto et al., 2009; Chen and Ge, 2010). A novel LAMP *in situ* detection method was reported for the rapid detection of food-borne *V. parahaemolyticus* strains, which has greater specificity and is less time-consumption than regular LAMP and other PCR-based methods (Wang et al., 2013a). Recently, Zeng et al. (2014) developed a novel method that combines the LAMP assay with immunomagnetic separation to detect *V. parahaemolyticus* in raw oysters. The limit of detection was 0.19 CFU/g, thus providing a platform for the comprehensive detection of pathogenic strains using a virulence- gene-specific LAMP assay (Zeng et al., 2014). Although LAMP is an effective and economic method to rapidly detect the pathogenic bacteria at one temperature without the need of cycling, however, similar to PCR, the methods of targeted separation and enrichments severally affected the application of LAMP.

### IMMUNOLOGICAL DETECTION

Immunological methods based on monoclonal antibodies are often used for the rapid detection and quantification of food-borne pathogens in seafood. Sandwich enzyme-linked immunosorbent assays based on monoclonal antibodies directed against TDH, TLH, and TRH have been used to identify these proteins in pathogenic clinical isolates of *V. parahaemolyticus* (Honda et al., 1989, 1990; Kumar et al., 2011; Sakata et al., 2012). However, these monoclonal antibodies do not detect all clinical and environmental *V. parahaemolyticus* strains because they cross-react with other bacteria (Prompamorn et al., 2013). An immunochromatographic assay was developed to detect the TDH hemolysin produced by *V. parahaemolyticus* in enrichment cultures from stool specimens (Kawatsu et al., 2006).

Today, recombinant antibody fragments, such as single-chain variable fragments (scFvs), have become an essential tool for research, diagnostic, and therapeutic purposes (Wang et al., 2014a). In 2012, our group has screened a high affinity scFv antibody successfully against a pathogenic factor TLH of *V. parahaemolyticus* by phage display. The screened scFv-LA3 antibody is specific to TLH antigen, and it is active against *Vibrio* cells possessing TLH (Wang et al., 2012). Our results indicated that scFv-LA3 recognizes specifically TLH produced by *V. parahaemolyticus* (Wang et al., 2014a), and it can be used as an antibody reagent to detect the TLH producing *V. parahaemolyticus* strains in seafood (Wang et al., 2012). Compared to the traditional full length Ig G antibody, the sensitivity of immunological method based on scFv is unsatisfactory, and the fact that current scFv antibodies have the poor stability, low solubility, and affinity seriously limits their diagnostic and clinic application. To improve the stability and solubility of scFv antibody, researchers have developed an Skp co-expressed system to express a functional scFv protein, and the Skp co-expressed scFv showed high solubility and binding activity to antigen TLH (Wang et al., 2013b).

### OTHERS METHODS

In addition to the methods discussed above, many detection methods based on biochemistry and biophysics have been used to detect and identify *V. parahaemolyticus* strains. As early as Su et al. (2005), a chromogenic medium was used for the selective and specific detection of *V. parahaemolyticus* strains. Hayashi et al. (2006), developed a novel method for the early detection of viable and TDH- or TRH-producing *V. parahaemolyticus* in seafood using soft-agar-coated filter combined with multiplex PCR, which identifies seafood samples contaminated with *V. parahaemolyticus* within 2 days. A new enrichment broth containing the bile salt, sodium taurocholate (ST broth) was used for improving the isolation and detection of pathogenic *V. parahaemolyticus* from seafood (Raghunath et al., 2009). A novel light-scattering sensor based solid agar plate has also been used for the real-time detection and identification of *V. parahaemolyticus*, *V. vulnificus*, and *V. cholerae* colonies (Huff et al., 2012). Dual-color flow cytometry was developed for the simultaneous detection of *V. parahaemolyticus* and *Salmonella typhimurium* in real samples. In this system, fluorescent quantum dots (QDs) labeled aptamers recognize the two bacterial species, and the sensitivity of detection was increased when QDs nanoparticles was used (Duan et al., 2013). Recently, Xiang et al. (2013) developed a real-time resistance measurement based on four different methods of detection *V. parahaemolyticus* by targeting the lecithin-dependent hemolysin gene: including LAMP, electrochemical ion bonding (crystal violet and Mg^2+^), real-time monitoring, and derivative analysis. The limit of detection was 10 CFU/mL, and the results revealed that this method is more accurate, sensitive, and specific than culture methods.

## PREVENTION AND CURES BASED ON VIRULENCE FACTORS

### ANTIBODY NEUTRALIZATION AND INHIBITION

Given the widespread contamination by *V. parahaemolyticus* and because it is strongly pathogenic to humans, it is very important to prevent and treat the diseases caused by this bacterium. However, to date, no effective measures are available to treat the diseases caused by *V. parahaemolyticus*, and the prospect of developing such therapies is still not good. Excitingly, antibody molecules have become extremely potent candidates for therapeutic applications, and have been developed into an important class of drugs for the treatment of numerous infectious diseases (Hagemeyer et al., 2009). An scFv antibody directed against the pathogenic factor TLH of *V. parahaemolyticus* effectively neutralized the cytotoxicity of *V. parahaemolyticus* TLH, thus exerting a protective effect on various types of TLH-infected cells (Wang et al., 2012). In *V. parahaemolyticus*, the needle complex is formed by the needle subunit protein (VP1694), which contains only 88 amino acids, and its function only relies on a single polymerized protein (Tamano, 2000; Blocker et al., 2008). The needle subunit may be a useful target protein for screening an effective antibody or inhibitor that can prevent the formation of the needle complex (Davis and Mecsas, 2007). A specific and high-affinity scFv antibody directed against VP1694 (needle subunit) has been prepared, and may play an important role in inhibiting the assembly of T3SS (Wang et al., 2014b). Significantly, the above results showed that the specific and functional scFv antibodies against target antigens of *V. parahaemolyticus* have been prepared successfully, and it provides a solid foundation for the immunological diagnosis and prevention of the diseases caused by *V. parahaemolyticus.*

### INHIBITOR-MEDIATED TARGETED THERAPIES

Type III secretion systems is conserved among different bacterial pathogens, and it may be an important potential therapeutic target (Gauthier et al., 2005; Mota et al., 2005). Like other Gram-negative pathogenic bacteria, *V. parahaemolyticus* contains a contact-dependent T3SS, which delivers several effectors into the cytosol of infected host cells (Makino et al., 2003; Park et al., 2004a; Sun et al., 2008; Worrall et al., 2011). The above description shows that T3SS might be a useful target for screening an effective inhibitor that can prevent the formation of the needle complex.

Many small chemical molecules have been shown to block assembly of T3SS, and some compounds broadly inhibit T3SS in many other bacterial pathogens (Izore et al., 2011). A high-throughput assay was developed to screen for a specific transcriptional inhibitor of the virulence factors in enteropathogenic *Escherichia coli*, to block the promoters of virulence associated factors and thus inhibit their transcription (Gauthier et al., 2005). Sulfonyl amino benzanilides and salicylidene anilides have been shown to inhibit the expression of T3SS- related genes, disrupting different pathways in enteropathogenic *E. coli* (Kauppi et al., 2003; Gauthier et al., 2005). Benzimidazoles also have also been shown to inhibit the transcription factors LcrF of *Yersinia pseudotuberculosis* and ExsA of *Pseudomonas aeruginosa* (Garrity-Ryan et al., 2010; Grier et al., 2010). Salicylidene acylhydrazide and thiazolidinone have been used to repress the formation and assembly of the needle complex, and to block the secretion of effectors in many bacterial pathogens, including *Shigella*, *Yersinia*, *Chlamydia,* and *Salmonella* spp. (Negrea et al., 2007; Veenendaal et al., 2009; Aiello et al., 2010). Other studies have suggested that thiazolidinones are multifaceted therapeutic agents for inhibiting bacterial infection (Dayam et al., 2006). In summary, the prevention and control of the diseases caused by *V. parahaemolyticus* mainly involve the use of antibiotics, or chemical molecules/drugs, but these inhibitors based on chemical molecules often lead to bacterial drug resistance or environmental residues of drug, resulting in enormous damage to the environment and human health (Pasqualinà et al., 2011; Silva et al., 2014b). Hence, it is very important to develop a feasible measure to improve this dilemma.

### BACTERIOPHAGE-BASED THERAPIES

The increasing prevalence of bacterial antibiotic resistance has prompted a search for candidate agents to replace antibiotics in the effective treatment of bacterial diseases. In recent years, therapies based on bacteriophages have become a topical issue in this field. With the development of phage biology research and genome sequencing, theses methods have been applied to the diagnosis and treatment of bacterial diseases (Laanto et al., 2012; Silva et al., 2014a). A mycobacteriophage delivered by a non-virulent *Mycobacterium* was reported to effectively kill the *M*. *avium* and *M*. *tuberculosis*, and has become a model for phage therapies directed against intracellular bacterial pathogens (Broxmeyer et al., 2002; Peng et al., 2006). The therapeutic efficacy of phage therapies has been demonstrated in many infectious diseases caused by members of the genus *Vibrio*, including *V. vulnificus*, *V. harveyi, V. parahaemolyticus,* and *V. anguillarum* (Shivu et al., 2007; Crothers-Stomps et al., 2010; Mateus et al., 2014). Phage therapy can protect against experimentally induced vibriosis in the Atlantic salmon, and can effectively prevent mortality during vibriosis of in the brine shrimp and *V. anguillarum* infections during the production of fish larvae (Higuera et al., 2013; Martinez-Diaz and Hipolito-Morales, 2013; Silva et al., 2014b). Unlike antibiotics or chemical drugs, phage therapies are inexpensive, and more environmentally friendly, and do not induce microbial resistance, suggesting that phage therapy is a suitable alternative treatment for vibriosis in aquaculture industries (Silva et al., 2014b). One of the main challenges in using bacteriophages to control pathogens in seafood is to control the efficacy and safety of phage, and the market acceptance of use of phage. The detailed characterization of phage properties and understanding of phage–host interactions are essential requirements for the successful application of phage-based pathogen control (Tan et al., 2014).

### CONTROLS AND PREVENTION

To reduce the risk of *V. parahaemolyticus* infections associated with seafood consumption, some strategies based on physical and chemical methods have been developed (Su and Liu, 2007). Thermal processing is a common approach to inactivating *V. parahaemolyticus* residues in seafood. Low-temperature freezing (at –18°C or –24°C) or high-temperature treatment (>55°C) for 10 min is reported to effectively inactivate or kill *V. parahaemolyticus* in oysters (Andrews et al., 2000). High-pressure processing (HPP) is another method that has also been used to destroy pathogenic microorganisms in seafood, and has been used extensively to inactivate *V. parahaemolyticus* in oysters (Calik et al., 2002; Cook et al., 2002; He et al., 2002). Irradiation is another important method of eliminating *V. parahaemolyticus* from oysters. It does not kill the oyster or alter its sensory qualities at low doses, but the safety issues associated with radioactive materials limits its use (Andrews et al., 2003; Jakabi et al., 2003). Similar to the approaches discussed above, chemical reagents have been developed to reduce the bacterial contamination in seafood, including chlorine, electrolyzed oxidizing water and iodophors (Croci et al., 2002; Ren and Su, 2006). However, none of these effectively dislodge *V. parahaemolyticus* from oysters, and further research is required that focuses on the screening and development of new drugs.

## CONCLUSION

*Vibrio parahaemolyticus* occurs naturally in marine, estuarine, and coastal environments throughout the world, and is the causative agent of food-borne gastroenteritis (Ceccarelli et al., 2013). The T3SSs are responsible for its cytotoxicity, and play a significant role in the induction of inflammatory chemokines in the host. T3SS1 is essential for systemic infection and the innate immune responses induced during intestinal infection, although the details of the mechanisms are still unclear, and the host targets remain to be determined (O’Boyle and Boyd, 2014). T3SS2 is associated with the enterotoxicity of *V. parahaemolyticus* in mammalian infection models *in vivo*, and has been reported to cause cytotoxicity in intestinal cell lines (Ham and Orth, 2012). The T6SSs, novel recently identified systems are necessary for the adhesion of *V. parahaemolyticus* to cells and are also involved in intracellular trafficking and vesicular transport. T6SS1 has antibacterial activity under warm conditions, enhancing the environmental fitness of *V. parahaemolyticus* (Salomon et al., 2013a), but our knowledge of the biological activity of T6SS2 is limited (Salomon et al., 2013a, 2014). Although a number of toxins and effectors associated with the pathogenesis of *V. parahaemolyticus* have been identified and characterized, but the detailed mechanisms of the total effectors of this bacterium, which have evolved to work together, and the distinct functions of individual effectors in causing pathogenicity are yet to be investigated. Further studies should focus on the correlation between T3SS and T6SS, and the non-invasive nature of *V. parahaemolyticus* warrants further investigation. Today, a large number of detection methods based on virulence factors are used for the detection and risk assessment of *V. parahaemolyticus.* However, to reduce the harm attributable to *V. parahaemolyticus,* specific, highly sensitive molecular methods are required to reliably identify and differentiate virulent and avirulent *V. parahaemolyticus* strains. The prevention and treatment of the diseases are still the key outcomes of future research, which should extend our understanding of the precise relationship between the disease in the human host and the pathogenicity of *V. Parahaemolyticus*. This review article provides insight into the control of the clinical risks posed by this potently virulent bacterium, which is extremely pathogenic to humans, by summarizing the molecular and therapeutic techniques available to future medical and immunological research. Effective control measures that combine novel drugs and targeted therapies must be developed to eradicate the risks posed to human health by this life-threatening disease exclusively.

## Conflict of Interest Statement

The authors declare that the research was conducted in the absence of any commercial or financial relationships that could be construed as a potential conflict of interest.

## References

[B1] Aiello D. Williams J. D. Majgier-Baranowska H. Patel I. Peet N. P. Huang J. (2010). Discovery and characterization of inhibitors of *Pseudomonas aeruginosa* type III secretion. *Antimicrob. Agents Chemother.* 54 1988–1999 10.1128/AAC.01598-09 20176902 PMC2863679

[B2] Alam M. J. Tomochika K. I. Miyoshi S. I. Shinoda S. (2002). Environmental investigation of potentially pathogenic *Vibrio parahaemolyticus* in the Seto-Inland Sea, Japan. *FEMS Microbiol. Lett.* 208 83–87 10.1111/j.1574-6968.2002.tb11064.x 11934498

[B3] Andrews L. Jahncke M. Mallikarjunan K. (2003). Low dose gamma irradiation to reduce pathogenic *Vibrio* in live oysters (*Crassostrea virginica*). *J. Aquat. Food Prod. Technol.* 12 71–82 10.1300/J030v12n03_07

[B4] Andrews L. S. Park D. L. Chen Y. P. (2000). Low temperature pasteurization to reduce the risk of *Vibrio* infections from raw shellstock oysters. *Food Addit. Contam.* 17 787–791 10.1080/026520300415336 11091792

[B5] Anonymous. (2012). *China Statistical Yearbook.* Beijing: State Statistical Bureau.

[B6] Barbieri J. T. Sun J. (2004). *Pseudomonas aeruginosa* ExoS and ExoT. *Rev. Physiol. Biochem. Pharmacol.* 152 79–92 10.1007/s10254-004-0031-7 15375697

[B7] Bej A. K. Patterson D. P. Brasher C. W. Vickery M. C. L. Jones D. D. Kaysner C. A. (1999). Detection of total and hemolysin-producing *Vibrio parahaemolyticus* in shellfish using multiplex PCR amplification of *tl*, *tdh* and *trh*. *J. Microbiol. Methods* 36 215–225 10.1016/S0167-7012(99)00037-8 10379807

[B8] Blake P. A. Merson M. H. Weaver R. E. Hollis D. G. Heublein P. C. (1979). Disease caused by a marine *Vibrio*. *Clin. Characterist. Epidemiol. N. Engl. J. Med.* 300 1–5 10.1056/NEJM197901043000101 758155

[B9] Blocker A. J. Deane J. E. Veenendaal A. K. Roversi P. Hodgkinson J. L. Johnson S. (2008). What’s the point of the type III secretion system needle? *Proc. Natl. Acad. Sci. U.S.A.* 105 6507–6513 10.1073/pnas.0708344105 18458349 PMC2373345

[B10] Boyd E. F. Cohen A. L. Naughton L. M. Ussery D. W. Binnewies T. T. Stine T. T. (2008). Molecular analysis of the emergence of pandemic *Vibrio parahaemolyticus*. *BMC Microbiol.* 8:110 10.1186/1471-2180-8-110 PMC2491623 18590559

[B11] Bresee J. S. Widdowson M. A. Monroe S. S. Glass R. I. (2002). Food-borne viral gastroenteritis: challenges and opportunities. *Clin. Infect. Dis.* 35 748–753 10.1086/342386 12203173

[B12] Broberg C. A. Calder T. J. Orth K. (2011). *Vibrio* parahaemolyticus cell biology and pathogenicity determinants. *Microbes Infect.* 13 992–1001 10.1016/j.micinf.2011.06.013 21782964 PMC3384537

[B13] Broberg C. A. Zhang L. Gonzalez H. Laskowski-Arce M. A. Orth K. (2010). A *Vibrio* effector protein is an inositol phosphatase and disrupts host cell membrane integrity. *Science* 329 1660–1662 10.1126/science.1192850 20724587

[B14] Broxmeyer L. Sosnowsk D. Miltner E. Chacón O. Wagner D. McGarvey J. (2002). Killing of *Mycobacterium avium* and *Mycobacterium tuberculosis* by a mycobacteriophage delivered by a nonvirulent *Mycobacterium*: a model for phage therapy of intracellular bacterial pathogens. *J. Infect. Dis.* 186 1155–1160 10.1086/343812 12355367

[B15] Burdette D. L. Seemann J. Orth K. (2009). *Vibrio* VopQ induces PI3-kinase-independent autophagy and antagonizes phagocytosis. *Mol. Microbiol.* 73 639–649 10.1111/j.1365-2958.2009.06798.x 19627496 PMC2733864

[B16] Burdette D. L. Yarbrough M. L. Orvedahl A. Gilpin C. J. Orth K. (2008). *Vibrio parahaemolyticus* orchestrates a multifaceted host cell infection by induction of autophagy, cell rounding, and then cell lysis. *Proc. Natl. Acad. Sci. U.S.A.* 105 12497–12500 10.1073/pnas.0802773105 18713860 PMC2527940

[B17] Cal Y. Ni Y. (1996). Purification, characterization, and pathogenicity of urease produced by *Vibrio parahaemolyticus*. *J. Clin. Lab. Anal.* 10 70–73 10.1002/(SICI)1098-2825612(1996)10:2<70::AID-JCLA2>3.0.CO;2-P 8852357

[B18] Calder T. Kinch L. N. Fernandez J. Salomon D. Grishin N. V. Orth K. (2014). *Vibrio* type III effector VPA1380 Is related to the cysteine protease domain of large bacterial toxins. *PLoS ONE* 9:e104387 10.1371/journal.pone.0104387 PMC4123922 25099122

[B19] Calik H. Morrissey M. T. Reno P. W. An H. (2002). Effect of high pressure processing on *Vibrio parahaemolyticus* strains in pure culture and *Pacific oysters*. *J. Food Sci.* 67 1506–1510 10.1111/j.1365-2621.2002.tb10313.x

[B20] Casselli T. Lynch T. Southward C. M. Jones B. W. Devinney R. (2008). *Vibrio parahaemolyticus* inhibition of Rho family GTPase activation requires a functional chromosome I type III secretion system. *Infect. Immun.* 76 2202–2211 10.1128/IAI.01704-07 18347050 PMC2346677

[B21] Ceccarelli D. Hasan N. A. Huq A. Colwell R. R. (2013). Distribution and dynamics of epidemic and pandemic *Vibrio parahaemolyticus* virulence factors. *Front. Cell Infect. Microbiol.* 3:97 10.3389/fcimb.2013.00097 PMC3858888 24377090

[B22] Chao G. X. Jiao X. N. Zhou X. H. Wang F. Yang Z. Q. Huang J. L. (2010). Distribution of genes encoding four pathogenicity Islands VPaIs, T6SS, Biofilm, and type I Pilus in food and clinical strains of *Vibrio parahaemolyticus* in China. *Foodborne Pathog. Dis.* 76 649–658 10.1089/fpd.2009.0441 20132020

[B23] Chao G. Jiao X. Zhou X. Yang Z. Huang J. Pan Z. (2009). Serodiversity, pandemic O3:K6 clone, molecular typing, and antibiotic susceptibility of foodborne and clinical *Vibrio parahaemolyticus* isolates in Jiangsu, China. *Foodborne Pathog. Dis.* 6 1021–1028 10.1089/fpd.2009.0295 19630509

[B24] Chatterjee S. Chaudhury S. McShan A. C. Kaur K. De Guzman R. N. (2013). Structure and biophysics of type III secretion in bacteria. *Biochemistry* 52 2508–2517 10.1021/bi400160a 23521714 PMC3711828

[B25] Chen M. B. Guo D. Wong H. C. Zhang X. Liu F. X. Chen H. Y. (2012). Development of O-serogroup specific PCR assay for detection and identification of *Vibrio parahaemolyticus*. *Int. J. Food Microbiol.* 159 122–129 10.1016/j.ijfoodmicro.2012.08.012 23072697

[B26] Chen S. Y. Ge B. L. (2010). Development of a toxR-based loop-mediated isothermal amplification assay for detecting *Vibrio parahaemolyticus*. *BMC Microbiol.* 10:41 10.1186/1471-2180-10-41 PMC2838873 20146814

[B27] Cook D. W. O’Leary P. Hunsucker J. C. Sloan E. M. Bowers J. C. Blodgett R. J. (2002). *Vibrio vulnificus* and *Vibrio parahaemolyticus* in US retail shell oysters: a national survey from June 1998 to July 1999. *J. Food Prot.* 65 79–87. 11808810 10.4315/0362-028x-65.1.79

[B28] Cornelis G. R. (2006). The type III secretion injectisome. *Nat. Rev. Microbiol.* 4 811–825 10.1038/nrmicro1526 17041629

[B29] Coulthurst S. J. (2013). The Type VI secretion system a wide spread and versatile cell targeting system. *Res. Microbiol.* 164 640–654 10.1016/j.resmic.2013.03.017 23542428

[B30] Croci L. Suffredini E. Cozzi L. Toti L. (2002). Effects of depuration of molluscs experimentally contaminated with *E. coli, Vibrio cholerae O*1 and *Vibrio parahaemolyticus*. *J. Appl. Microbiol.* 92 460–465 10.1046/j.1365-2672.2002.01548.x 11872121

[B31] Crothers-Stomps C. Høj L. Bourne D. G. Hall M. R. Owens L. (2010). Isolation of lytic bacteriophage against *Vibrio harveyi*. *J. Appl. Microbiol.* 108 1744–1750 10.1111/j.1365-2672.2009.04578.x 19886890

[B32] Cruz C. D. Hedderley D. Fletcher G. C. (2015). *Vibrio parahaemolyticus* prevalence and distribution in New Zealand shellfish: a long-term study. *Appl. Environ. Microbiol.* 10.1128/AEM.04020-14 [Epub ahead of print]. PMC4357935 25616790

[B33] Daniels N. A. Ray B. Easton A. Marano N. Kahn E. McShan A. L. (2000). Emergence of a new O3:K6 V. parahaemolyticus serotype in raw oysters. *J. Am. Med. Assoc.* 284 1541–1545 10.1001/jama.284.12.1541 11000648

[B34] Davis A. J. Mecsas J. (2007). Mutations in the *Yersinia pseudotuberculosis* type III secretion system needle protein, Ysc F, that specifically abrogate effector translocation into host cells. *J. Bacteriol.* 189 83–97 10.1128/JB.01396-06 17071752 PMC1797200

[B35] Dayam R. Aiello F. Deng J. Wu Y. Garofalo A. Chen X. (2006). Discovery of small molecule integrin alphavbeta3 antagonists as novel anticancer agents. *J. Med. Chem.* 49 4526–4534 10.1021/jm051296s 16854058

[B36] Dean P. (2011). Functional domains and motifs of bacterial type III effector proteins and their roles in infection. *FEMS Microbiol. Rev.* 35 1100–1125 10.1111/j.1574-6976.2011.00271.x 21517912

[B37] DePaola A. Kaysner C. A. Bowers J. Cook D. W. (2000). Environmental investigations of *Vibrio parahaemolyticus* in oysters after outbreaks in Washington, Texas, and New York (1997 and 1998). *Appl. Environ. Microbiol.* 66 4649–4654 10.1128/AEM.66.11.4649-4654.2000 11055906 PMC92362

[B38] Duan N. Wua S. J. Yu Y. Ma X. Y. Xia Y. Chen X. J. (2013). A dual-color flow cytometry protocol for the simultaneous detection of *Vibrio parahaemolyticus* and *Salmonella typhimurium* using aptamer conjugated quantum dots as labels. *Anal. Chim. Acta* 804 151–158 10.1016/j.aca.2013.09.047 24267076

[B39] Fraylick J. E. Rucks E. A. Greene D. M. Vincent T. S. Olson J. C. (2002). Eukaryotic cell determination of Exo S ADP-ribosyltransferase substrate specificity. *Biochem. Biophys. Res. Commun.* 291 91–100 10.1006/bbrc.2002.6402 11829467

[B40] Friebel A. Ilchmann H. Aepfelbacher M. Ehrbar K. Machleidt W. Hardt W. D. (2001). Sop E and Sop E2 from *Salmonella typhimurium* activate different sets of Rho GTPases of the host cell. *J. Biol. Chem.* 276 34035–34040 10.1074/jbc.M100609200 11440999

[B41] Fujino T. Okuno Y. Nakada D. Aoyama A. Mukai T. Ueho T. (1953). On the bacteriological examination of shirasu food poisoning. *Med. J. Osaka Univ.* 4 299–304.

[B42] Garrido A. Chapela M. J. Ferreira M. Atanassova M. Fajardo P. Lago J. (2012). Development of a multiplex real-time PCR method for pathogenic *Vibrio parahaemolyticus* detection (*tdh*^+^ and *trh*^+^). *Food Control.* 24 128–135 10.1016/j.foodcont.2011.09.015

[B43] Garrity-Ryan L. K. Kim O. K. Balada-Llasat J. M. Bartlett V. J. Verma A. K. Fisher M. L. (2010). Small molecule Inhibitors of LcrF, a *Yersinia pseudotuberculosis* transcription factor, attenuate virulence and limit infection in a murine pneumonia model. *Infect. Immun.* 78 4683–4690 10.1128/IAI.01305-09 20823209 PMC2976336

[B44] Gauthier A. Robertson M. L. Lowden M. Ibarra J. A. Puente J. L. Finlay B. B. (2005). Transcriptional inhibitor of virulence factors in enteropathogenic *Escherichia coli*. *Antimicrob. Agents Chemother.* 49 4101–4109 10.1128/AAC.49.10.4101-4109.2005 16189086 PMC1251496

[B45] Geissler B. (2012). Bacterial toxin effector-membrane targeting: outside in, then back again. *Front. Cell Infect. Microbiol.* 2:75 10.3389/fcimb.2012.00075 PMC3417404 22919666

[B46] Gode-Potratz C. J. Kustusch R. J. Breheny P. J. Weiss D. S. McCarter L. L. (2011). Surface sensing in *Vibrio parahaemolyticus* triggers a programme of gene expression that promotes colonization and virulence. *Mol. Microbiol.* 79 240–263 10.1111/j.1365-2958.2010.07445.x 21166906 PMC3075615

[B47] Gonzalez-Escalona N. Strain E. A. De Jesus A. J. Jones J. L. Depaola A. (2011). Genome sequence of a clinical O4:K12 serotype *Vibrio parahaemolyticus* strain 10329. *J. Bacteriol.* 193 3405–3406 10.1128/JB.05044-11 21551294 PMC3133290

[B48] Gotoh K. Kodama T. Hiyoshi H. Izutsu K. Park K.-S. Dryselius R. (2010). Bile acid-induced virulence gene expression of *Vibrio parahaemolyticus* reveals a novel therapeutic potential for bile acid sequestrants. *PLoS ONE* 5:e13365 10.1371/journal.pone.0013365 PMC2954181 20967223

[B49] Grier M. C. Garrity-Ryan L. K. Bartlett V. J. Klausner K. A. Donovan P. J. Dudley C. (2010). *N*-Hydroxybenzimidazole inhibitors of ExsA MAR transcription factor in *Pseudomonas aeruginosa*: in vitro anti-virulence activity and metabolic stability. *Bioorg. Med. Chem. Lett.* 20 3380–3383 10.1016/j.bmcl.2010.04.014 20434913

[B50] Hagemeyer C. E. Von Zur Muhlen C. von Elverfeldt T. Peter K. (2009). Single-chain antibodies as diagnostic tools and therapeutic agents. *J. Thromb. Haemost.* 101 1012–1019 10.1160/TH08-12-0816 19492141

[B51] Haglund C. M. Welch M. D. (2011). Pathogens and polymers: microbe-host interactions illuminate the cytoskeleton. *J. Cell Biol.* 195 7–17 10.1083/jcb.201103148 21969466 PMC3187711

[B52] Ham H. Orth K. (2012). The role of type III secretion system 2 in *Vibrio parahaemolyticus* pathogenicity. *J. Microbiol.* 50 719–725 10.1007/s12275-012-2550-2 23124738

[B53] Hayashi S. Okura M. Osawa R. (2006). Soft-agar-coated filter method for early detection of viable and thermostable direct hemolysin (TDH)- or TDH-related hemolysin-producing *Vibrio parahaemolyticus* in seafood. *Appl. Environ. Microbiol.* 72 4576–4582 10.1128/AEM.02646-05 16820446 PMC1489382

[B54] He H. Adams R. M. Farkas D. F. Morrissey M. T. (2002). Use of high pressure processing for oyster shucking and shelf-life extension. *J. Food Sci.* 67 640–644 10.1111/j.1365-2621.2002.tb10652.x

[B55] Hicks S. W. Galan J. E. (2013). Exploitation of eukaryotic subcellular targeting mechanisms by bacterial effectors. *Nat. Rev. Microbiol.* 11 316–326 10.1038/nrmicro3009 23588250 PMC3859125

[B56] Higa N. Toma C. Koizumi Y. Nakasone N. Nohara T. Masumoto J. (2013). *Vibrio parahaemolyticus* effector proteins suppress inflammasome activation by interfering with host autophagy signaling. *PLoS Pathog.* 9:e1003142 10.1371/journal.ppat.1003142 PMC3554609 23357873

[B57] Higuera G. Bastias R. Tsertsvadze G. Romero J. Espejo R. T. (2013). Recently discovered *Vibrio anguillarum* phages can protect against experimentally induced *vibrio*sis in Atlantic salmon, *Salmo salar*. *Aquaculture* 395 128–133 10.1016/j.aquaculture.2013.02.013

[B58] Hiyoshi H. Kodama T. Iida T. Honda T. (2010). Contribution of *Vibrio parahaemolyticus* virulence factors to cytotoxicity, enterotoxicity, and lethality in mice. *Infect. Immun.* 78 1772–1780 10.1128/IAI.01051-09 20086084 PMC2849405

[B59] Hiyoshi H. Kodama T. Saito K. Gotoh K. Matsuda S. Akeda Y. (2011). Vop V, an F-actin binding type III secretion effector, is required for *Vibrio parahaemolyticus* induced enterotoxicity. *Cell Host Microbe* 10 401–409 10.1016/j.chom.2011.08.014 22018240

[B60] Hlady W. G. Klontz K. C. (1996). The epidemiology of *Vibrio* infections in Florida, 1981–1993. *J. Infect. Dis.* 173 1176–1183 10.1093/infdis/173.5.1176 8627070

[B61] Ho B. T. Dong T. G. Mekalanos J. J. (2014). A view to a kill: the bacterial type VI secretion system. *Cell Host Microbe* 15 9–21 10.1016/j.chom.2013.11.008 24332978 PMC3936019

[B62] Honda T. Iida T. (1993). The pathogenicity of *Vibrio* parahaemolyticus and the role of the thermostable direct heamolysin and related heamolysins. *Rev. Med. Microbiol.* 4 106–113 10.1097/00013542-199304000-00006

[B63] Honda T. Ni Y. X. Miwatani T. (1988). Purification and characterization of a hemolysin produced by a clinical isolate of Kanagawa phenomenon negative *Vibrio parahaemolyticus* and related to the thermostable direct hemolysin. *Infect. Immun.* 56 961–965. 3126151 10.1128/iai.56.4.961-965.1988 PMC259398

[B64] Honda T. Ni Y. X. Miwatani T. (1990). Production of monoclonal antibodies against haemolysin (Vp-TRH) produced by *Vibrio parahaemolyticus*. *FEMS Microbiol. Lett.* 56 167–170 10.1111/j.1574-6968.1990.tb04143.x 2332155

[B65] Honda T. Ni Y. Yoh M. Miwatani T. (1989). Production of monoclonal antibodies against thermostable direct haemolysin of *Vibrio parahaemolyticus* and application of the monoclonal antibodies for enzyme link immunosorbent assay. *Med. Microbiol. Immunol.* 178 245–253 10.1007/BF00191059 2779485

[B66] Hondo S. Goto I. Minematsu I. Ikeda N. Asano N. Ishibashi M. (1987). Gastroenteritis due to Kanagawa negative *Vibrio parahaemolyticus*. *Lancet* 1 331–332 10.1016/S0140-6736(87)92062-9 2880146

[B67] Hossain M. T. Kim Y. O. Kong I. S. (2013). Multiplex PCR for the detection and differentiation of *Vibrio parahaemolyticus* strains using the *groEL, tdh* and *trh* genes. *Mol. Cell Probe.* 27 171–175 10.1016/j.mcp.2013.04.001 23660458

[B68] Huff K. Aroonnual A. Littlejohn A. E. F. Rajwa B. Bae E. Banada P. P. (2012). Light-scattering sensor for real-time identification of *Vibrio parahaemolyticus*, *Vibrio vulnificus* and *Vibrio cholerae* colonies on solid agar plate. *Microb Biotechnol.* 5 607–620 10.1111/j.1751-7915.2012.00349.x 22613192 PMC3815873

[B69] Izore T. Job V. Dessen A. (2011). Biogensis, regulation, an targeting of the type III secretion system. *Cell Press* 19 603–612 10.1016/j.str.2011.03.015 21565695

[B70] Izumiya H. Matsumoto K. Yahiro S. Lee J. Morita M. Yamamoto S. (2011). Multiplex PCR assay for identification of three major pathogenic *Vibrio* spp., *Vibrio cholerae, Vibrio parahaemolyticus, and Vibrio vulnificus. Mol. Cell. Probe* 25 174–176 10.1016/j.mcp.2011.04.004 21530641

[B71] Jakabi M. Gelli D. S. Torre J. C. M. D. Rodas M. A. B. Franco B. D. G. M. Destro M. T. (2003). Inactivation by ionizing radiation of *Salmonella enteritidis*, *Salmonella infantis*, and *Vibrio parahaemolyticus* in oyster (*Crassostrea brasiliana*). *J. Food Prot.* 66 1025–1029. 12801004 10.4315/0362-028x-66.6.1025

[B72] Jegga A. G. Schneider L. Ouyang X. Zhang J. (2011). Systems biology of the autophagy-lysosomal pathway. *Autophagy* 7 477–489 10.4161/auto.7.5.14811 21293178 PMC3127210

[B73] Jones J. L. Lüdeke C. H. Bowers J. C. DeRosia-Banick K. Carey D. H. Hastback W. (2014). Abundance of *Vibrio cholerae*, V. *vulnificus,* and V. *parahaemolyticus* in oysters (*Crassostrea virginica*) and clams (*Mercenaria mercenaria*) from Long Island sound. *Appl. Environ. Microbiol.* 80 7667–7672 10.1128/AEM.02820-14 25281373 PMC4249230

[B74] Jones J. L. Ludeke C. H. Bowers J. C. Garrett N. Fischer M. Parsons M. B. (2012). Biochemical, serological, and virulence characterization of clinical and oyster *Vibrio parahaemolyticus* isolates. *J. Clin. Microbiol.* 50 2343–2352 10.1128/JCM.00196-12 22535979 PMC3405591

[B75] Joseph S. Colwell R. Kaper J. (1982). *Vibrio parahaemolyticus* and related halophilic *Vibrios*. *Crit. Rev. Microbiol.* 10 77–124 10.3109/10408418209113506 6756788

[B76] Kajino-Sakamoto R. Inagaki M. Lippert E. Akira S. Robine S. Matsumoto K. (2008). Enterocyte-derived TAK1 signaling prevent sepithelium apoptosis and the development of ileitis and colitis. *J. Immunol.* 181 1143–1152 10.4049/jimmunol.181.2.1143 18606667 PMC3065656

[B77] Kauppi A. M. Nordfelth R. Uvell H. Wolf-Watz H. Elofsson M. (2003). Targeting bacterial virulence: inhibitors of type III secretion in *Yersinia*. *Chem. Biol.* 10 241–249 10.1016/S1074-5521(03)00046-2 12670538

[B78] Kawatsu K. Ishibashi M. Tsukamoto T. (2006). Development and evaluation of a rapid, simple, and sensitive immunochromatographic assay to detect thermostable direct hemolysin produced by *Vibrio parahaemolyticus* in enrichment cultures of stool specimens. *J. Clin. Microbiol.* 44 1821–1827 10.1128/JCM.44.5.1821-1827.2006 16672412 PMC1479187

[B79] Kim J.-J. Jo E.-K. (2013). NLRP3 inflammasome and host protection against bacterial infection. *J. Korean Med. Sci.* 28 1415–1423 10.3346/jkms.2013.28.10.1415 24133343 PMC3792593

[B80] Kodama T. Rokuda M. Park K. S. Cantarelli V. V. Matsuda S. Iida T. (2007). Identification and characterization of Vop T, a novel ADP- ribosyltransferase effector protein secreted via the *Vibrio* parahaemolyticus type III secretion system 2. *Cell Microbiol.* 9 2598–2609 10.1111/j.1462-5822.2007.00980.x 17645751

[B81] Kodama T. Yamazaki C. Park K. S. Akeda Y. Iida T. Honda T. (2010). Transcription of *Vibrio parahaemolyticus* T3SS1 genes is regulated by a dual regulation system consisting of the ExsACDE regulatory cascade and H-NS. *FEMS Microbiol. Lett.* 311 10–17 10.1111/j.1574-6968.2010.02066.x 20722736

[B82] Krauss M. Haucke V. (2007). Phosphoinositides: regulators of membrane traffic and protein function. *FEBS Lett.* 581 2105–2111 10.1016/j.febslet.2007.01.089 17316616

[B83] Kumar B. K. Raghunah P. Devegowda D. Deekshit K. Venugopal N. V. Karunasagar I. (2011). Development of monoclonal antibody based sandwich ELISA for the rapid detection of pathogenic *Vibrio parahaemolyticus* in seafood. *Intl. J. Food microbiol.* 145 244–249 10.1016/j.ijfoodmicro.2010.12.030 21276628

[B84] Laanto E. Bamford J. K. H. Laakso J. Sundberg L. R. (2012). Phage-driven loss of virulence in a fish pathogenic bacterium. *PLoS ONE* 7:e53157 10.1371/journal.pone.0053157 PMC3534065 23308090

[B85] Lee J. K. Jung D. W. Eom S. Y. Oh S. W. Kim Y. J. Kwak H. S. (2008). Occurrence of *Vibrio parahaemolyticus* in oysters from Korean retail outlets. *Food Control* 19 990–994 10.1016/j.foodcont.2007.10.006

[B86] Levin R. E. (2006). *Vibrio parahaemolyticus*, a notably lethal human pathogen derived from seafood: a review of its pathogenicity, characteristics, subspecies characterization, and molecular methods of detection. *Food Biotechnol.* 20 93–128 10.1080/08905430500524275

[B87] Letchumanan V. Chan K. G. Lee L. H. (2014). *Vibrio parahaemolyticus*: a review on the pathogenesis, prevalence, and advance molecular identification techniques. *Front. Microbiol.* 5:705 10.3389/fmicb.2014.00705 PMC4263241 25566219

[B88] Letchumanan V. Yin W. F. Lee L. H. Chan K. G. (2015). Prevalence and antimicrobial susceptibility of *Vibrio parahaemolyticus* isolated from retail shrimps in Malaysia. *Front Microbiol* 6:33 10.3389/fmicb.2015.00033 PMC4311705 25688239

[B89] Liu H. (2003). Analysis of the collective food poisoning events in Shanghai from 1990 to 2000. *Chinese J. Nat. Med.* 5 17–20.

[B90] Liu X. M. Chen Y. Fan Y. X. Wang M. Q. (2006). Foodborne diseases occurred in 2003 report of the National Foodborne Diseases Surveillance system, China. *Wei Sheng Yan Jiu* 35 201–204. 16758972

[B91] Liverman A. D. Cheng H. C Trosky J. E. Leung D. W. Yarbrough M. L. Burdette D. L. (2007). Arp2/3-independent assembly of actin by *Vibrio* type III effector Vop L. *Proc. Natl. Acad. Sci. U.S.A.* 104 17117–17122 10.1073/pnas.0703196104 17942696 PMC2040399

[B92] Lozano-León A. Torres J. Osorio C. R. Martínez-Urtaza J. (2003). Identification of tdh-positive *Vibrio parahaemolyticus* from an outbreak associated with raw oyster consumption in Spain. *FEMS Microbiol. Lett.* 226 281–284 10.1016/S0378-1097(03)00604-9 14553923

[B93] Luong P. Kinch L. N. Brautigam C. A. Grishin N. V. Tomchick D. R. Orth K. (2010). Kinetic and structural insights into the mechanism of AMPylation by Vop S Fic domain. *J. Biol. Chem.* 285 20155–20163 10.1074/jbc.M110.114884 20410310 PMC2888428

[B94] Makino K. Oshima K. Kurokawa K. Yokoyama K. Uda T. Tagomori K. (2003). Genome sequence of *Vibrio parahaemolyticus*: a pathogenic mechanism distinct from that of V cholerae. *Lancet* 361 743–749 10.1016/S0140-6736(03)12659-1 12620739

[B95] Martinez-Diaz S. F. Hipolito-Morales A. (2013). Efficacy of phage therapy to prevent mortality during the *vibrio*sis of brine shrimp. *Aquaculture* 400 120–124 10.1016/j.aquaculture.2013.03.007

[B96] Martinez-Urtaza J. Simental L. Velasco D. DePaola A. Ishibashi M. Nakaguchi Y. (2005). Pandemic *Vibrio parahaemolyticus* O3:K6, Europe. *Emerg. Infect. Dis. J.* 11 1319–1320 10.3201/eid1108.050322 PMC3320470 16110585

[B97] Mateus C. Costa C. Silva Y. Cunha A. Almeida A. (2014). Efficiency of phage cocktails in the inactivation of *Vibrio* in aquaculture. *Aquaculture* 424 167–173 10.1016/j.aquaculture.2014.01.001

[B98] Matlawska-Wasowska K. Finn R. Mustel A. O’Byrne C. P. Baird A. W. Coffey E. T. (2010). The *Vibrio parahaemolyticus* type III secretion systems manipulate host cell MAPK for critical steps in pathogenesis. *BMC Microbiol.* 10:329 10.1186/1471-2180-10-329 PMC3022711 21192810

[B99] Matsuda S. Kodama T. Okada N. Okayama K. Honda T. Iida T. (2010). Association of *Vibrio parahaemolyticus* thermostable direct hemolysin with lipid rafts is essential for cytotoxicity but not hemolytic activity. *Infect. Immun.* 78 603–610 10.1128/IAI.00946-09 19933828 PMC2812206

[B100] Matsuda S. Okada N. Kodama T. Honda T. Iida T. (2012). A cytotoxic type III secretion effector of *Vibrio parahaemolyticus* targets vacuolar H^+^-ATPase subunit c and ruptures host cell lysosomes. *PLoS Pathog.* 8:e1002803 10.1371/journal.ppat.1002803 PMC3400558 22829766

[B101] McCarthy S. A. DePaola A. Cook D. W. Kaysner C. A. Hill W. E. (1999). Evaluation of alkaline phosphatase- and digoxigenin-labelled probes for detection of the thermolabile hemolysin (*tlh*) gene of *Vibrio parahaemolyticus*. *Lett. Appl. Microbiol.* 28 66–70 10.1046/j.1365-2672.1999.00467.x 10030035

[B102] Meador C. E. Parsons M. M. Bopp C. A. Gerner-Smidt P. Painter J. A. Vora G. J. (2007). Virulence gene and pandemic group-specific marker profiling of clinical *Vibrio parahaemolyticus* isolates. *J. Clin. Microbiol.* 45 1133–1139 10.1128/JCM.00042-07 17301274 PMC1865801

[B103] Miyamoto Y. Kato T. Obara Y. Akiyama S. Takizawa K. Yamai S. (1969). In vitro hemolytic characteristic of *Vibrio parahaemolyticus*: its close correlation with human pathogenicity. *J. Bacteriol.* 100 1147–1149. 5391048 10.1128/jb.100.2.1147-1149.1969 PMC250216

[B104] Mota L. J. Sorg I. Cornelis G. R. (2005). Type III secretion: the bacteria-eukaryotic cell express. *FEMS Microbiol. Lett.* 252 1–10 10.1016/j.femsle.2005.08.036 16216444

[B105] Nair G. B Ramamurthy T. Bhattacharya S. K. Dutta B. Takeda Y. Sack D. A. (2007). Global dissemination of *Vibrio parahaemolyticus* serotype O3:K6 and its serovariants. *Clin. Microbiol. Rev.* 20 39–48 10.1128/CMR.00025-06 17223622 PMC1797631

[B106] Nakaguchi Y. (2013). Contamination by *Vibrio parahaemolyticus* and its virulent strains in seafood marketed in Thailand, Vietnam, Malaysia, and Indonesia. *Trop Med. Health* 41 95–102 10.2149/tmh.2011-06 24155650 PMC3800702

[B107] Namgoong S. Boczkowska M. Glista M. J. Winkelman J. D. Rebowski G. Kovar D. R. (2011). Mechanism of actin filament nucleation by *Vibrio* VopL and implications for tandem-W domain nucleation. *Nat. Struct. Mol. Biol.* 18 1060–1067 10.1038/nsmb.2109 21873985 PMC3173040

[B108] Negrea A. Bjur E. Ygberg S. E. Elofsson M. Wolf-Watz H. Rhen M. (2007). *Salicylidene acylhydrazides* that affect type III protein secretion in *Salmonella enterica* serovar typhimurium. *Antimicrob. Agents Chemother.* 51 2867–2876 10.1128/AAC.00223-07 17548496 PMC1932493

[B109] Nemoto J. Sugawara C. Akahane K. Hashimoto K. Kojima T. Ikedo M. (2009). Rapid and specific detection of the thermostable direct hemolysin gene in *Vibrio parahaemolyticus* by loop mediated isothermal amplification. *J. Food Prot.* 72 748–754. 19435222 10.4315/0362-028x-72.4.748

[B110] Nishibuchi M. Fasano A. Russell R. G. Kaper J. B. (1992). Enterotoxigenicity of *Vibrio parahaemolyticus* with and without genes encoding thermostable direct hemolysin. *Infect. Immun.* 60 3539–3545. 1500161 10.1128/iai.60.9.3539-3545.1992 PMC257358

[B111] Nishibuchi M. Kaper J. B. (1995). Thermostable direct hemolysin gene of *Vibrio parahaemolyticus*: a virulence gene acquired by a marine bacterium. *Infect. Immun.* 63 2093–2099. 7768586 10.1128/iai.63.6.2093-2099.1995 PMC173271

[B112] Nordstrom J. L. Vickery M. C. L. Blackstone G. M. Murray S. L. DePaola A. (2007). Development of a multiplex real-time PCR assay with an internal amplification control for the detection of total and pathogenic *Vibrio parahaemolyticus* bacteria in oysters. *Appl. Environ. Microbiol.* 73 5840–5847 10.1128/AEM.00460-07 17644647 PMC2074920

[B113] O’Boyle N. Boyd A. (2014). Manipulation of intestinal epithelial cell function by the cell contact-dependent typeIII secretion systems of *Vibrio parahaemolyticus*. *Front. Cell Infect. Microbiol.* 3:114 10.3389/fcimb.2013.00114 PMC3887276 24455490

[B114] Okada N. Matsuda S. Matsuyama J. Park K. S. de los Reyes C. Kogure K. (2010). Presence of genes for type III secretion system 2 in *Vibrio mimicus* strains. *BMC Microbiol.* 10:302 10.1186/1471-2180-10-302 PMC3004890 21110901

[B115] Ono T. Kwon-Sam P. Ueta M. Iida T. Honda T. (2006). Identification of proteins secreted via *Vibrio parahaemolyticus* type III secretion system 1. *Infect. Immun.* 74 1032–1042 10.1128/IAI.74.2.1032-1042.2006 16428750 PMC1360304

[B116] Osawa R. Okitsu T. Morozumi H. Yamai S. (1996). Occurrence of urease-positive *Vibrio parahaemolyticus* in Kanagawa, Japan, with specific reference to presence of thermostable direct hemolysin (TDH) and the TDH-related hemolysin genes. *Appl. Environ. Microbiol.* 62 725–727. 8593076 10.1128/aem.62.2.725-727.1996 PMC167841

[B117] Paranjpye R. Hamel O. S. Stojanovski A. Liermann M. (2012). Genetic diversity of clinical and environmental *Vibrio parahaemolyticus* strains from the Pacific Northwest. *Appl. Environ. Microbiol.* 78 8631–8638 10.1128/AEM.01531-12 23042162 PMC3502901

[B118] Park K. S. Ono T. Rokuda K. Jang M. H. Okada K. Iida T. (2004a). Functional characterization of two type III secretion systems of *Vibrio parahaemolyticus*. *Infect. Immun.* 72 6659–6665 10.1128/IAI.72.11.6659-6665.2004 15501799 PMC523034

[B119] Park K. S. Ono T. Rokuda M. Jang M. H. Iida T. Honda T. (2004b). Cytotoxicity and enterotoxicity of the thermostable direct hemolysin-deletion mutants of *Vibrio parahaemolyticus*. *Microbiol. Immunol.* 48 313–318 10.1111/j.13480421 15107542

[B120] Park K. S. Ono T. Rokuda M. Jang M. H. Okada K. Iida T. (2004c). Functional characterization of two III secretion systems of *Vibrio parahaemolyticus*. *Infect. Immun.* 72 6659–6665 10.1128/IAI.72.11.6659-6665.2004 15501799 PMC523034

[B121] Pasqualinà L. Gabriella C. Eleonora M. Renata Z. Santi D. (2011). Susceptibility to antibiotics of *Vibrio* spp. and *Photobacterium damselae* ssp. piscicida strains isolated from Italian aquaculture farms. *New Microbiol.* 34 53–63. 21344147

[B122] Peng F. M. Jiang D. Y. Ruan H. H. Liu H. Q. Zhou L. P. (2010). Pathogenic investigation on a food poisoning induced by *Vibrio parahaemolyticus*. *Prev. Med. Trib.* 16 746–747.

[B123] Peng L. Chen B. W. Luo Y. A. Wang G. Z. (2006). Effect of mycobacteriophage to intracellular mycobateria in vitro. *Chin. Med. J.* 119 692–695. 16635416

[B124] Porta H. Cancino-Rodezno A. Soberon M. Bravo A. (2011). Role of MAPK p38 in the cellular responses to pore-forming toxins. *Peptides* 32 601–606 10.1016/j.peptides.2010.06.012 20599578 PMC2994946

[B125] Prochazkova K. Satchell K. J. (2008). Structure-function analysis of inositol hexakisphosphate-induced auto processing of the *Vibrio cholerae* multifunctional auto processing RTX toxin. *J. Biol. Chem.* 283 23656–23664 10.1074/jbc.M803334200 18591243 PMC3259750

[B126] Prompamorn P. Longyant S. Pengsuk C. Sithigorngul P. Chaivisuthangkura P. (2013). Rapid identification and differentiation of *Vibrio parahaemolyticus* from *Vibrio* spp. in seafood samples using developed monoclonal antibodies. *World J. Microbiol. Biotechnol.* 29 721–731 10.1007/s11274-012-1228-6 23233121

[B127] Pruitt R. N. Chagot B. Cover M. Chazin W. J. Spiller B. Lacy D. B. (2009). Structure function analysis of inositol hexakisphosphate-induced auto processing in *Clostridium difficile* toxin A. *J. Biol. Chem.* 284 21934–21940 10.1074/jbc.M109.018929 19553670 PMC2755918

[B128] Pukatzki S. Ma A. T. Revel A. T. Sturtevant D. Mekalanos J. J. (2007). Type VI secretion system translocates a phage tail spike-like protein into target cells where it cross-links actin. *Proc. Natl. Acad. Sci. U.S.A.* 104 15508–15513 10.1073/pnas.0706532104 17873062 PMC2000545

[B129] Pukatzki S. Ma A. T. Sturtevant D. Krastins B. Sarracino D. Nelson W. C. (2006). Identification of a conserved bacterial protein secretion system in *Vibrio cholerae* using the Dictyostelium host model system. *Proc. Natl. Acad. Sci. U.S.A.* 103 1528–1533 10.1073/pnas.0510322103 16432199 PMC1345711

[B130] Qi J. Du Y. Zhu R. Zhu X. Bai H. Hu M. (2012). A loop-mediated isothermal amplification method for rapid detection of the multidrug-resistance gene cfr. *Gene* 504 140–143 10.1016/j.gene.2012.04.049 22579470

[B131] Raghunath P. Karunasagar I. Karunasagar I. (2009). Improved isolation and detection of pathogenic *Vibrio parahaemolyticus* from seafood using a new enrichment broth. *Int. J. Food Microbiol.* 129 200–203 10.1016/j.ijfoodmicro.2008.11.026 19103467

[B132] Rahimi E. Ameri M. Doosti A. Gholampour A. R. (2010). Occurrence of toxigenic *Vibrio parahaemolyticus* strains in shrimp in Iran. *Foodborne Pathog. Dis.* 7 1107–1111 10.1089/fpd.2010.0554 20528175

[B133] Ren T. Su Y. C. (2006). Effects of electrolyzed oxidizing water treatment on reducing *Vibrio parahaemolyticus* and *Vibrio vulnificus* in raw oysters. *J. Food Prot.* 69 1829–1834. 16924906 10.4315/0362-028x-69.8.1829

[B134] Ritchie J. M. Rui H. Bronson R. T. Waldor M. K. (2010). Back to the future: studying cholera pathogenesis using infant rabbits. *mBio* 1:e00047-10 10.1128/mBio.00047-10 PMC2912669 20689747

[B135] Ritchie J. M. Rui H. Zhou X. Iida T. Kodoma T. Ito S. (2012). Inflammation and disintegration of intestinal villi in an experimental model for *Vibrio parahaemolyticus* induced diarrhea. *PLoS Pathog* 8:e1002593 10.1371/journal.ppat.1002593 PMC3305451 22438811

[B136] Robert-Pillot A. Copin S. Gay M. Malle P. Quilici M. L. (2010). Total and pathogenic *Vibrio parahaemolyticus* in shrimp: fast and reliable quantification by real-time PCR. *Int. J. Food Microbiol.* 143 190–197 10.1016/j.ijfoodmicro.2010.08.016 20843573

[B137] Rosec J. P. Simon M. Causse V. Boudjemaa M. (2009). Detection of total and pathogenic *Vibrio parahaemolyticus* in shellfish: comparison of PCR protocols using pR72H or toxR targets with a culture method. *Int. J. Food Microbiol.* 129 136–145 10.1016/j.ijfoodmicro.2008.11.017 19106014

[B138] Sakata J. Kawatsu K. Kawahara R. Kanki M. Iwasaki T. Kumeda Y. (2012). Production and characterization of a monoclonal antibody against recombinant thermolabile hemolysin and its application to screen for *Vibrio parahaemolyticus* contamination in raw seafood. *Food Control* 23 171–176 10.1016/j.foodcont.2011.07.005

[B139] Sakazaki R. Tamura K. Kato T. Obara Y. Yamai S. (1968). Studies on the enteropathogenic, facultatively halophilic bacterium, *Vibrio parahaemolyticus.* 3. Enteropathogenicity. *Jpn. J. Med. Sci. Biol.* 21 325–331 10.7883/yoken1952.21.325 4886581

[B140] Salomon D. Guo Y. R. Kinch L. N. Grishin N. V. Gardner K. H. Orth K. (2013a). Effectors of animal and plant pathogens use a common domain to bind host phosphoinositides. *Nat. commun.* 4:2973 10.1038/ncomms3973 PMC4981085 24346350

[B141] Salomon D. Gonzalez H. Updegraff B. L. Orth K. (2013b). *Vibrio parahaemolyticus* type VI secretion system 1 is activated in marine conditions to target bacteria, and is differentially regulated from system 2. *PLoS ONE* 8:e61086 10.1371/journal.pone.0061086 PMC3628861 23613791

[B142] Salomon D. Kinch L. N. Trudgian D. C. Guo X. F. Klimko J. A. Grishin N. V. (2014). Marker for type VI secretion system effectors. *Proc. Natl. Acad. Sci. U.S.A.* 111 9271–9276 10.1073/pnas.1406110111 24927539 PMC4078801

[B143] Shimohata T. Nakano M. Lian X. Shigeyama T. Iba H. Hamamoto A. (2011). *Vibrio parahaemolyticus* infection induces modulation of IL-8 secretion through dual pathway via VP1680 in Caco-2 cells. *J. Infect. Dis.* 203 537–544 10.1093/infdis/jiq070 21177635 PMC3071237

[B144] Shimohata T. Takahashi A. (2010). Diarrhea induced by infection of *Vibrio parahaemolyticus*. *J. Med. Invest.* 57 179–182 10.2152/jmi.57.179 20847516

[B145] Shinoda S. Matsuoka H. Tsuchie T. Miyoshi S. Yamamoto S. Taniguchi H. (1991). Purification and characterization of a lecithin-dependent haemolysin from *Escherichia coli* transformed by a *Vibrio parahaemolyticus* gene. *J. Gen. Microbiol.* 137 2705–2711 10.1099/00221287-137-12-2705 1791426

[B146] Shivu M. M. Rajeeva B. C. Girisha S. K. Karunasagar I. Krohne G. Karunasagar I. (2007). Molecular characterization of *Vibrio harveyi* bacteriophages isolated from aquaculture environments along the coast of India. *Environ. Microbiol.* 9 322–331 10.1111/j.1462-2920.2006.01140.x 17222131

[B147] Shneider M. M. Buth S. A. Ho B. T. Basler M. Mekalanos J. J. Leiman P. G. (2013). PAAR-repeat proteins sharpen and diversify the type VI secretion system spike. *Nature* 500 350–353 10.1038/nature12453 23925114 PMC3792578

[B148] Silva Y. J. Costa L. Pereira C. Cunha A. Calado R. Gomes N. C. (2014a). Influence of environmental variables in the efficiency of phage therapy in aquaculture. *Microb. Biotechnol.* 7 401–413 10.1111/1751-7915.12090 24841213 PMC4229321

[B149] Silva Y. J. Costa L. L. Pereira C. Mateus C. Cunha A. Calado R. (2014b). Phage therapy as an approach to prevent *Vibrio anguillarum* infections in fish larvae production. *PLoS ONE* 9:e114197 10.1371/journal.pone.0114197 PMC4252102 25464504

[B150] Sreelatha A. Bennett T. L. Zheng H. Jiang Q. X. Orth K. Starai V. J. (2013). *Vibrio* effector protein, Vop Q, forms a lysosomal gated channel that disrupts host ion homeostasis and autophagic flux. *Proc. Natl. Acad. Sci. U.S.A.* 110 11559–11564 10.1073/pnas.1307032110 23798441 PMC3710849

[B151] Su Y. C. Duan J. Wu W. H. (2005). Selectivity and specificity of a chromogenic medium for detecting *Vibrio parahaemolyticus*. *J. Food Prot.* 68 1454–1456. 16013386 10.4315/0362-028x-68.7.1454

[B152] Su Y. C. Liu C. C. (2007). *Vibrio parahaemolyticus*: a concern of seafood safety. *Food microbiol.* 24 549–558 10.1016/j.fm.2007.01.005 17418305

[B153] Sun P. Tropea J. E. Austin B. P. Cherry S. Waugh D. S. (2008). Structural characterization of the *Yersinia pestis* type III secretion needle protein YscF in complex with its heterodimeric chaperone YscE/YscG. *J. Mol. Biol.* 377 819–830 10.1016/j.jmb.2007.12.067 18281060 PMC2329918

[B154] Takahashi A. Kenjyo N. Imura K. Myonsun Y. Honda T. (2000). Cl- secretion in colonic epithelial cells induced by the *Vibrio parahaemolyticus* hemolytic Toxin related to thermostable direct hemolysin. *Infect. Immun.* 68 5435–5438 10.1128/IAI.68.9.5435-5438.2000 10948178 PMC101812

[B155] Tamano K. (2000). Supra molecular structure of the *Shigella* type III secretion machinery: the needle part is changeable in length and essential for delivery of effectors. *EMBO J.* 19 3876–3887 10.1093/emboj/19.15.3876 10921870 PMC306602

[B156] Tan D. Gram L. Middelboe M. (2014). *Vibrio*phages and their interactions with the fish pathogen *Vibrio anguillarum*. *Appl. Environ. Microbiol.* 80 3128–3140 10.1128/AEM.03544-3513 24610858 PMC4018917

[B157] Tran L. Nunan L. Redman R. M. Mohney L. L. Pantoja C. R. Fitzsimmons K. (2013). Determination of the infectious nature of the agent of acute hepatopancreatic necrosis syndrome affecting penaeid shrimp. *Dis. Aquat. Organ.* 105 45–55 10.3354/dao02621 23836769

[B158] Trosky J. E. Mukherjee S. Burdette D. L. Roberts M. Mccarter L. Siegel R. M. (2004). Inhibition of MAPK signaling pathways by Vop A from *Vibrio parahaemolyticus*. *J. Biol. Chem.* 279 51953–51957 10.1074/jbc.M407001200 15459200

[B159] Tyagi A. Saravanan V. Karunasagar I. Karunasagar I. (2009). Detection of *Vibrio parahaemolyticus* in tropical shellfish by SYBR green real-time PCR and evaluation of three enrichment media. *Int. J. Food Microbiol.* 129 124–130 10.1016/j.ijfoodmicro.2008.11.006 19106013

[B160] Veenendaal A. K. J. Sundin C. Blocker A. J. (2009). Small-molecule type III secretion system inhibitors block assembly of the *Shigella* type III secreton. *J. Bacteriol.* 191 563–570 10.1128/JB.01004-08 18996990 PMC2620818

[B161] Wang R. Z. Huang J. D. Zhang W. Lin G. M. Lian J. W. Jiang L. B. (2011a). Detection and identification of *Vibrio parahaemolyticus* by multiplex PCR and DNA-DNA hybridization on a microarray. *J. Genet. Genomics* 38 129–135 10.1016/j.jgg.2011.02.002 21477785

[B162] Wang H. P. Zhang J. L. Jiang T. Bao Y. X. Zhou X. M. (2011b). Insufficiency of the Kanagawa hemolytic test for detecting pathogenic *Vibrio parahaemolyticus* in *Shanghai*, China. *Diagn. Microbiol. Infect. Dis.* 69 7–11 10.1016/j.diagmicrobio.2010.08.016 21146708

[B163] Wang L. Shi L. Su J. Y. Ye Y. X. Zhong Q. P. (2013a). Detection of *Vibrio parahaemolyticus* in food samples using in situ loop-mediated isothermal amplification method. *Gene* 515 421–425 10.1016/j.gene.2012.12.039 23266627

[B164] Wang R. Z. Xiang S. S. Feng Y. J. Srinivas S. Zhang Y. F. Lin M. S. (2013b). Engineering production of functional scFv antibody in *E. coli* by co-expressing the molecule chaperone Skp. *Front. Cell Infect. Microbiol.* 3:72 10.3389/fcimb.2013.00072 PMC3818579 24224158

[B165] Wang R. Z. Fang S. Wu D. L. Wu D. Lian J. Fan J. (2012). Screening of a ScFv antibody that can neutralize effectively the cytotoxicity of *Vibrio parahaemolyticus* TLH. *Appl. Environ. Microbiol.* 78 4967–4975 10.1128/AEM.00435-12 22562997 PMC3416367

[B166] Wang R. Z. Xiang S. S. Zhang Y. H. Chen Q. Y. Zhong Y. F. Wang S. H. (2014a). Development of a functional antibody by using a green fluorescent protein frame as the template. *Appl. Environ. Microbiol.* 80 4126–4137 10.1128/AEM.00936-14 24795367 PMC4068687

[B167] Wang R. Z. Fang S. Xiang S. S. Ling S. M. Yuan J. Wang S. H. (2014b). Generation and Characterization of a scFv Antibody Against T3SS Needle of *Vibrio parahaemolyticus*. *Indian J. Microbiol.* 54 143–150 10.1007/s12088-013-0428-426 25320414 PMC4188505

[B168] Ward L. N. Bej A. K. (2006). Detection of *Vibrio parahaemolyticus* in shellfish by use of multiplexed real-time PCR with TaqMan fluorescent probes. *Appl. Environ. Microbiol.* 72 2031–2042 10.1128/AEM.72.3.2031-2042.2006 16517652 PMC1393209

[B169] Worrall L. J. Lameignere E. Strynadka N. C. (2011). Structural overview of the bacterial injectisome. *Curr. Opin. Microbiol.* 14 3–8 10.1016/j.mib.2010.10.009 21112241

[B170] Xiang G. M. Pu X. Y. Jiang D. N. Liu L. L. Liu C. Liu X. B. (2013). Development of a real-time resistance measurement for *Vibrio parahaemolyticus* detection by the Lecithin-Dependent hemolysin gene. *PLoS ONE* 8:e72342 10.1371/journal.pone.0072342 PMC3753338 23991096

[B171] Xu X. K. Wu Q. P. Zhang J. M. Cheng J. H. Zhang S. H. Wu K. (2014). Prevalence, pathogenicity, and serotypes of *Vibrio parahaemolyticus* in shrimp from Chinese retail markets. *Food Control* 46 81–85 10.1016/j.foodcont.2014.04.042

[B172] Yamazaki W. Ishibashi M. Kawahara R. Inoue K. (2008). Development of a loopmediated isothermal amplification assay for sensitive and rapid detection of *Vibrio parahaemolyticus*. *BMC Microbiol.* 8:163 10.1186/1471-2180-8-163 PMC2572065 18823567

[B173] Yarbrough M. L. Li Y. Kinch L. N. Grishin N. V. Ball H. L. Orth K. (2009). AMPylation of RhoGTPases by *Vibrio* Vop S disrupts effector binding and downstream signaling. *Science* 323 269–272 10.1126/science.1166382 19039103

[B174] Yeung P. S. Boor K. J. (2004). Epidemiology, pathogenesis, and prevention of foodborne *Vibrio parahaemolyticus* infections. *Foodborne Pathog. Dis.* 1 74–88 10.1089/153531404323143594 15992266

[B175] Yu B. Cheng H.-C. Brautigam C. A. Tomchick D. R. Rosen M. K. (2011). Mechanism of actin filament nucleation by the bacterial effector VopL. *Nat. Struct. Mol. Biol.* 18 1068–1074 10.1038/nsmb.2110 21873984 PMC3168117

[B176] Yu Y. Yang H. Li J. Zhang P. Wu B. Zhu B. (2012). Putative type VI secretion systems of *Vibrio parahaemolyticus* contribute to adhesion to cultured cell monolayers. *Arch. Microbiol.* 194 827–835 10.1007/s00203-012-0816-z 22535222

[B177] Zeng J. Wei H. Y. Zhang L. Liu X. F. Zhang H. Y. Cheng J. X. (2014). Rapid detection of *Vibrio parahaemolyticus* in raw oysters using immunomagnetic separation combined with loop-mediated isothermal amplification. *Int. J. Food Microbiol.* 174 123–128 10.1016/j.ijfoodmicro.2014.01.004 24480190

[B178] Zhang L. Krachler A. M. Broberg C. A. Li Y. Mirzaei H. Gilpin C. J. (2012). Type III effector Vop C mediates invasion for *Vibrio* species. *Cell Rep.* 1 453–460 10.1016/j.celrep.2012.04.004 22787576 PMC3392014

[B179] Zhang L. L. Orth K. (2013). Virulence determinants for *Vibrio parahaemolyticus* infection. *Curr. Opin. Microbiol.* 16 1–8 10.1016/j.mib.2013.02.002 23433802

[B180] Zhang X. H. Austin B. (2005). Haemolysins in *Vibrio* species. *J. Appl. Microbiol.* 98 1011–1019 10.1111/j.1365-2672.2005.02583.x 15836469

[B181] Zhao X. Li Y. Wang L. You L. Xu Z. Li L. (2011). Development and application of a loop-mediated isothermal amplification method on rapid detection of *Pseudomonas aeruginosa* strains. *World J. Microbiol. Biotechnol.* 27 181–184 10.1007/s11274-010-0429-0

[B182] Zhou X. Gewurz B. E. Ritchie J. M. Takasaki K. Greenfeld H. Kieff E. (2013). A *Vibrio parahaemolyticus* T3SS effector mediates pathogenesis by independently enabling intestinal colonization and inhibiting TAK1 activation. *Cell Rep.* 3 1690–1702 10.1016/j.celrep.2013.03.039 23623501 PMC3711673

[B183] Zhou X. Konkel M. E. Call D. R. (2010). Regulation of type III secretion system 1 gene expression in *Vibrio parahaemolyticus* is dependent on interactions between ExsA, ExsC, and ExsD. *Virulence* 1 260–272 10.4161/viru.1.4.12318 21178451 PMC3073295

[B184] Zhu R. G. Li T. P. Jia Y. F. Song L. F. (2012). Quantitative study of viable *Vibrio parahaemolyticus* cells in raw seafood using propidium monoazide in combination with quantitative PCR. *J. Microbiol. Methods* 90 262–266 10.1016/j.mimet.2012.05.019 22677606

