# Corrigendum: The pathogenesis, detection, and prevention of *Vibrio parahaemolyticus*

**DOI:** 10.3389/fmicb.2015.00437

**Published:** 2015-05-21

**Authors:** Rongzhi Wang, Yanfang Zhong, Xiaosong Gu, Jun Yuan, Abdullah F. Saeed, Shihua Wang

**Affiliations:** Key Laboratory of Biopesticide and Chemical Biology of Education Ministry and Key Laboratory of Pathogenic Fungi and Mycotoxins of Fujian Province, School of Life Sciences, Fujian Agriculture and Forestry UniversityFuzhou, China

**Keywords:** *Vibrio parahaemolyticus*, pathogenesis, virulence factors, detection, prevention

Page 5, “T6SS and pathogenesis” section.

1. “The T6SS organelle is functionally analogous to T3SS, and may have a critical function in the process of bacterial infection (Salomon et al., 2013a).” (Left, page 5, line 15).

correct to “The T6SS organelle **is supposed to be** functionally analogous to T3SS, and may have a critical function in the process of *V. parahaemolyticus* adhesion.

2. “T6SS2 has been found in both clinical and environmental isolates, is encoded on **chromosome I**,” (Left, page 5, line 22).

correct to “T6SS2 has been found in both clinical and environmental isolates, is encoded on **chromosome II**,”

3. The sentence “The VP1415 is another effector for T6SS1, and it is sufficient to exert toxicity (Salomon et al., 2014).” should be added at the last of this section. (Right, page 5, line 13).

## Conflict of interest statement

The authors declare that the research was conducted in the absence of any commercial or financial relationships that could be construed as a potential conflict of interest.

